# Successfully Navigating Food and Drug Administration Orphan Drug and Rare Pediatric Disease Designations for AAV9-hPCCA Gene Therapy: The National Institutes of Health Platform Vector Gene Therapy Experience

**DOI:** 10.1089/hum.2022.232

**Published:** 2023-03-20

**Authors:** Richa Madan Lomash, Oleg Shchelochkov, Randy J. Chandler, Charles P. Venditti, Anne R. Pariser, Elizabeth A. Ottinger

**Affiliations:** ^1^ Therapeutic Development Branch, Division of Preclinical Innovation, National Center for Advancing Translational Sciences (NCATS), NIH, Rockville, Maryland, USA; ^2^ Organic Acid Research Section, Molecular Medicine Branch, National Human Genome Research Institute (NHGRI), NIH, Bethesda, Maryland, USA; ^3^ Division of Rare Diseases Research Innovation, NCATS, NIH, Rockville, Maryland, USA.

**Keywords:** rare diseases, orphan drug, orphan drug designation, Rare Pediatric Disease Designation, financial incentives, gene therapy

## Abstract

Orphan drug designation (ODD) is an important program intended to facilitate the development of orphan drugs in the United States. An orphan drug benefiting pediatric patients can qualify as a drug for a Rare Pediatric Disease Designation (RPDD) as well. The ODD and RPDD programs provide financial incentives for development of diagnostic drugs, preventive measures, and treatment of diseases affecting small patient populations (adult and pediatric) for which commercial development would otherwise be very challenging. In 2019, a multidisciplinary group of collaborators at National Institutes of Health (NIH) embarked upon a gene therapy platform program called Platform Vector Gene Therapy (PaVe-GT) intended to develop gene therapies for four such rare disorders. An important part of PaVe-GT is to publicly share scientific and regulatory experience gained at different stages during the implementation of the PaVe-GT platform utilizing illustrative examples. The PaVe-GT team recently obtained ODD and RPDD for an adeno-associated virus gene therapy to treat propionic acidemia. Given an increasing interest in obtaining ODD for gene therapy, especially by small companies, research investigators, and patient groups, we overview the submission process and subsequently provide examples of our ODD and RPDD applications. Our ODD and RPDD applications and templates can also be found on the PaVe-GT website. Shared reference documents will have great utility to assist parties who may have limited experience with the preparation of similar applications for their orphan product.

## INTRODUCTION: THE PLATFORM VECTOR GENE THERAPY PROGRAM

In 2019, the National Center for Advancing Translational Sciences (NCATS) at the National Institutes of Health (NIH) embarked on a multiplexed approach to facilitate the standardization of gene therapy development of rare genetic diseases called the Platform Vector Gene Therapy (PaVe-GT) program. The main goal of PaVe-GT is to test whether the efficiency of gene therapy development and clinical implementation can be increased using shared approaches to streamline manufacturing, preclinical studies, and clinical protocols to treat rare monogenic diseases. PaVe-GT has been described in more detail elsewhere,^1^ but briefly, this program uses an adeno-associated virus (AAV) as a “platform” vector to develop gene therapies for four very low prevalence genetic diseases for which there would otherwise be no or little commercial interest.

The diseases selected in PaVe-GT include two organic acidemias, propionyl-CoA carboxylase alpha subunit propionic acidemia (*PCCA*-related PA) and isolated methylmalonic acidemia cobalamin type B (*MMAB*-related MMA), and two congenital myasthenic syndromes caused by deficiency of DOK7 (downstream of tyrosine kinase 7) or Collagen Q (ColQ), a form of nonfibrillar collagen. PaVe-GT also intends to help the rare disease community better understand the regulatory requirements and opportunities related to late-stage preclinical through early clinical development for investigational gene therapy drug products and ways to navigate the regulatory process. Our aim is to leverage the learnings and experience of PaVe-GT to other AAV gene therapy development programs, irrespective of the capsid or disease type. As part of this effort, the program plans to release a series of regulatory documentation and communications throughout our drug development program. Additional program information and project updates can be found on the PaVe-GT website (https://pave-gt.ncats.nih.gov/).

The first gene therapy product, adeno-associated virus serotype 9 human propionyl-CoA carboxylase, alpha subunit (AAV9-hPCCA, NCATS-BL0746) for the treatment of *PCCA-*related PA, was granted an Orphan Drug Designation (ODD) by the U.S. Food and Drug Administration (FDA) in 2021^2^ and subsequently a Rare Pediatric Disease Designation (RPDD) as a drug for the treatment of a rare pediatric disease, *PCCA-*related PA in 2022. The focus of this communication is to help readers understand the significance of these incentive programs in stimulating drug development and illustrate how sponsors can utilize FDA guidance to prepare ODD or RPDD applications. A sponsor, as per FDA regulations (21CFR 312.3), is defined as a person who takes responsibility for and initiates a clinical investigation. The sponsor may be a government agency, academic institution, pharmaceutical company, private organization, or an individual.^3^

## BACKGROUND: THE ORPHAN DRUG ACT, ODD, AND RPDD

It is estimated that there are more than 7,000 rare diseases,^4,5^ the majority of which are single gene disorders.^5,6^ While the number of people with an individual rare disease is small, the total number of individuals living with a rare disease in the United States is estimated at 25–30 million.^7^ Most rare diseases (∼95%) do not have an FDA-approved therapy,^8^ highlighting the considerable need for new treatments for this relatively large cohort of patients. Since an individual rare disease patient population is small, drug profitability is likely to be low in comparison to the cost of drug development, leading to a low return on investment for a company that develops “orphan drugs.” The Orphan Drug Act (ODA), enacted in the United States in 1983, supports the development of orphan drugs (including biologics) for rare diseases or conditions by providing financial incentives, thus reducing the overall development costs.^9^ The ODA is administered by FDA's Office of Orphan Product Development (OOPD),^10^ and they have issued regulations detailing requirements for granting an orphan designation.^11^

For a drug to qualify as an orphan drug,^9,11^ it must:

(a) Be intended for the treatment of a disease or condition affecting fewer than a total of 200,000 people in the United States, or
(b) If the drug is a vaccine, diagnostic drug, or preventive drug, the persons to whom the drug will be administered in the United States must be fewer than 200,000 per year, or
(c) For drugs intended for a disease or condition affecting 200,000 or more people, or for a vaccine, diagnostic drug, or preventive drug to be administered to 200,000 or more persons per year in the United States, the drug may qualify as an orphan drug when there is no reasonable expectation the cost of developing such a drug would be profitable by sales in the United States.

There are substantial financial benefits to obtaining an ODD^12^ that include:

(a) Tax credits for qualified clinical testing upon approval,
(b) Waiver of New Drug Application (NDA) or Biologics Licensing Application (BLA) Prescription Drug User Fee Act (PDUFA) fees (∼$3.1 M in 2022)^10^ upon submission of the application,
(c) Eligibility for 7-year marketing exclusivity, termed “orphan exclusivity,” postmarketing approval.^9,13,14^

Another program to incentivize therapeutic invention, the Priority Review Vouchers (PRVs) program, originally established for neglected tropical diseases, was subsequently expanded to include rare pediatric diseases.^15^ The RPDD program was started in 2012 and extended by the Congress in 2020^16^ with the intention of encouraging the development of drugs for rare pediatric disorders. RPDD can be used alone or in combination with other incentive programs, like ODD. A RPDD is for a serious or life-threatening rare disease or condition primarily affecting individuals from birth to 18 years. If a drug qualifies under a RPDD, and receives marketing approval, a sponsor is eligible to receive a PRV that may be used for an expedited review of a subsequent marketing application for a different product or sold to another sponsor.^17^ According to Government Accounting Office (GAO) report, 31 PRVs (19 for rare pediatric diseases) were awarded until 2019; of these, 16 vouchers have been redeemed thus far by sponsors for several indications.^18^

## ODD REQUEST OVERVIEW

In this article, we share our experience for successfully navigating the process of obtaining an ODD and a RPDD for an AAV9 gene therapy to treat *PCCA-*related PA. We submitted our applications separately, but the ODD and RPDD applications may be submitted simultaneously. Both applications typically rely upon similar information, so submitting together can streamline the process and timeline. Since we submitted the ODD first, we will primarily outline the process using our ODD example.

An ODD can be requested for a new drug, without any prior regulatory approval for treatment, prevention, or diagnosis of a rare disease or condition or for a previously approved drug if it is intended to treat, prevent, or diagnose a rare disease or condition that is different from the prior marketing approval. In addition, before drug approval, more than one sponsor may receive ODD for the same drug and the same rare disease or condition, but each sponsor seeking ODD must file a complete request for ODD, and the first sponsor to receive FDA drug approval receives the financial development benefits and conditional marketing exclusivity.^11,19^ Similarly, a marketing application is conditionally designated as a RPDD with the final determination made at the time of drug approval.^17^

According to the recent FDA guidance, “Interpreting Sameness of Gene Therapy Products Under the Orphan Drug Regulations,”^20^ FDA generally intends to determine whether two gene therapy products are different drugs on a case-by-case basis based on the principal molecular structural features, such as transgenes and vectors (capsids), for purposes of 21 CFR 316.3(b)(14)(ii). Usually, two AAV gene therapy products are considered different if they express a different transgene or have different vectors which could impact the tropism, immune response avoidance, or potential insertional mutagenesis. However, two gene therapy products are not considered to be different drugs based solely on minor differences between their transgenes and/or vectors. If two gene therapy products express the same transgene and have the same vector, determination of their sameness may also depend on additional features of the final product that can contribute to the therapeutic effect such as regulatory elements (*e.g.*, promoters, enhancers, and so on).

A sponsor may request ODD at any time in its drug development process (preclinical through clinical phases) before submission of a marketing application.^21^ It is, however, necessary to identify the lead investigational drug product for treating the orphan disease/condition before preparing the request since ODDs are specific to drug products. The information available can vary per program and any evidence that helps better describe the context of use of the proposed drug and its benefit in the disease indication can support an ODD application. This evidence can be in the form of literature, expert opinion, disease models, basic and translational studies, or reports.

Among the barriers that families and small patient advocacy groups face, the establishment of direct connections with researchers, clinicians, and disease experts for navigating disease information and estimating the patient population size are challenging. NCATS Division of Rare Diseases Research Innovation has resources such as the Genetic and Rare Diseases Information Center (GARD), Patient-toolkit, and Rare Diseases Clinical Research Network (RDCRN) to help groups further their understanding of the disease and make connections with appropriate experts. Scientific meetings organized by patient groups and patient focused conferences are also effective means to directly interact with researchers and clinicians. Once the right connections are made, sponsors may work with disease experts and drug developers to draft their own ODD application or outsource drafting of their application to commercial vendors providing such services.

**Figure 1.**
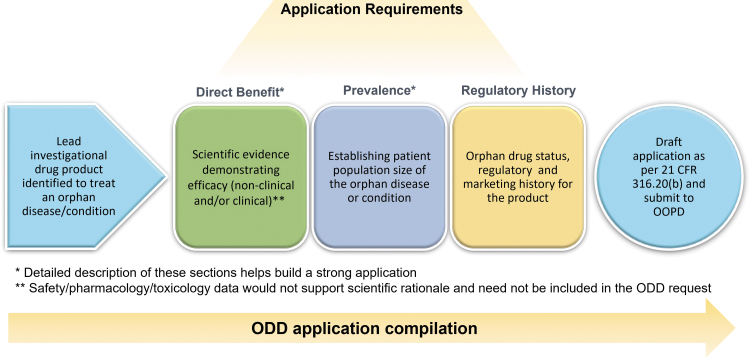
The ODD application compilation process. The application can be compiled as per the FDA requirements at different program stages once the lead investigational drug product has been identified. The application must include proof of direct benefit, documentation of population size, and orphan drug regulatory history. Direct benefit information and population size are critical, and substantial details in these sections make a strong application. FDA, Food and Drug Administration; ODD, orphan drug designation.

An overview of the ODD process is outlined in Fig. 1 with the main considerations being:

- Proof of direct benefit: Establishing a medically plausible basis for the drug's effectiveness in the rare disease supported by efficacy data from either clinical or nonclinical studies in a relevant model of the human disease.
- Patient population size in the United States: Establishing the orphan status of the disease or condition by determining population size from relevant literature and/or appropriate sources.

As required by the OOPD, we drafted a comprehensive ODD request application and submitted it to FDA OOPD, with all the information outlined under 21 CFR 316.20 “Content, and format of a request for ODD.”^22^ As a tool intended to assist sponsors in providing the ODD request information completely and succinctly, FDA OOPD provides the optional Form FDA 4035 “FDA Orphan Drug Designation Request Form,” where the requirements for an ODD request have been included.^23^ Alternatively, sponsors may compile their own application based on FDA regulatory requirements. We compiled our own application and, during the process, created a checklist to serve as a guide (Table 1) and prepared a template (available on PaVe-GT website). The lightly redacted ODD and RPDD applications that were submitted to the FDA are also provided (Supplementary Files S1 and S2).

**Table 1.** Orphan drug designation request checklist as per Food and Drug Administration regulations

Required Section of the ODD Request		Description of the Requirements
□	ODD request statement	A statement from the sponsor requesting an ODD for an investigational drug product for a rare disease or condition. 21 CFR 316.20(b)(1)
□	Administrative information	Name and address of the sponsor, sponsor's primary and any alternate contact(s), and regulatory agent(s) (where applicable). 21 CFR 316.20(b)(2)
□	Description of the rare disease or condition, proposed indication and use of the drug, and the reasons why such therapy is needed	A description of the rare disease or condition for which the investigational drug is being or will be investigated for, the proposed indication or indications for use of the drug, and the reasons why such therapy is needed. 21 CFR 316.20(b)(3)
□	Description of the drug and scientific rationale for use	Detailed description of investigational product; its physical and chemical properties, if these characteristics can be determined; and a discussion of the scientific rationale, based either on nonclinical or clinical efficacy data, to establish a medically plausible basis for the use of the drug for the rare disease or condition. 21 CFR 316.20(b)(4)
□	Orphan drug status	Only applicable to the sponsor of a drug that is otherwise the same drug as an already approved drug seeks ODD for the subsequent drug for the same rare disease or condition. If this is not applicable, provide a statement accordingly in the application under this section. 21 CFR 316.20(b)(5)
□	Patient subset considerations and medical plausibility of the chosen subset	Only applicable to the sponsor of an ODD request for an investigational drug for only a subset of persons with a particular disease or condition that otherwise affects 200,000 or more people (“orphan subset”). If this is not applicable, provide a statement accordingly in the application under this section. 21 CFR 316.20(b)(6)
□	Regulatory status and marketing history	This section should include a summary of the regulatory status and marketing history of the drug in the United States and in foreign countries. Include short description and assigned regulatory submission number to any historical regulatory submissions. 21 CFR 316.20(b)(7)
□	Documentation of patient population size	Provide documentation that the investigational drug is either intended to treat a disease or condition affecting fewer than a total of 200,000 people in the United States (prevalence); the investigational drug is intended to treat an acute disease or condition affecting annually fewer than 200,000 people in the United States (incidence); or, for a disease or condition affecting more than 200, 000 in the United States, provide documentation that there is no reasonable expectation that costs of research and development of the drug for the indication can be recovered by sales of the drug in the United States. 21 CFR 316.20(b)(8)

ODD, orphan drug designation.

*Source:* 21CFR 316, Subpart C—Designation of an Orphan Drug.^22^

## APPROACH: CONTENT OF AN ODD APPLICATION

### ODD request statement

The application request starts with a statement from the sponsor and the following is what we used for our application. “Pursuant to 21 CFR 316.20, National Institutes of Health (NIH), National Center for Advancing Translational Sciences (NCATS) requests designation of adeno-associated virus serotype 9 human propionyl-CoA carboxylase, alpha subunit (AAV9-hPCCA) as an orphan drug product for treatment of patients with propionic acidemia (PA) resulting from a deficiency of propionyl-CoA carboxylase, alpha subunit (PCCA).” See Section 1 in Supplementary File S1 for the complete statement.

### Description of the rare disease or condition, proposed indication and use of the drug, and the reasons why such therapy is needed

The ODD application requires that an orphan disease or condition targeted by the proposed product be clearly described. To the extent possible, it is useful to describe the clinical manifestations, pathophysiology, clinical course (*i.e.*, natural history), affected population (age range, age of onset, and demographics), medical care and therapies available to patients, disease mechanisms, and molecular underpinnings of the disease.

For *PCCA*-related PA, we reviewed published literature and gathered expert opinions and information from an NIH-conducted natural history study (NHS).^24,25^ We summarized the abovementioned sources to define the disease (*PCCA*-related PA) and included information pertinent to its clinical manifestations and pathophysiology. Briefly, PA is an autosomal recessive inborn error of metabolism for which the affected enzyme, PCC, a ubiquitously expressed mitochondrial enzyme, and its metabolic pathway for amino- and fatty-acid catabolism are known.^26–28^ PCC is a multimeric enzyme, composed of alpha (PCCA) and beta (PCCB) subunits, deficiency in either of which can result in *PCCA*- and *PCCB*-related PA with identical clinical manifestations,^29^ and pathogenic variants in either subunit occur with equal frequency. Each form of PA (*PCCA-* and *PCCB*-related) is a life-threatening chronic condition, which usually first manifests in the newborn period. Chronic manifestations of the disease are often punctuated by episodes of metabolic decompensation.

Currently available therapies incompletely control the disease, and there are no FDA-approved drugs for the treatment of PA. It should be noted that the ODD was requested specifically for the *PCCA*-related PA only, as the gene replacement therapy targeting the alpha subunit (AAV9-hPCCA) would only be expected to address the *PCCA*-related form of PA and not have any effect on PA caused by pathogenic variants in the *PCCB* gene (Section 3.1 in Supplementary File S1).

### Description of the drug and scientific rationale relevant to the disease/condition

The drug's description, including active ingredient, drug class/type, structure, physical and chemical properties, route of administration (ROA), formulation, and manufacturer details, is of value to the ODD request. ODD is generally conferred to the active moiety rather than the product formulation; therefore, changes to the product formulation should not generally affect ODD status.^30^

Data demonstrating that the investigational drug product has the “potential” or “promise” to treat, prevent, or diagnose the intended disease or condition are critical. Evidence supporting the scientific rationale, drug's mechanism of action, and its relevance to the disease/condition and efficacy data through clinical and/or nonclinical studies must be included. In cases where clinical data and appropriate animal models don't exist, *in vitro* data along with supporting information such as drug's mechanism of action, disease pathogenesis, and how the data relate to the disease may be presented.^31–33^

The gene therapy drug, AAV9-hPCCA, is an AAV9 vector expressing a functional human codon optimized complementary DNA (cDNA) encoding *PCCA*. The AAV9-hPCCA will deliver a functional copy of the human *PCCA* gene, allow for expression of wild-type PCCA protein, and help restore the PCC enzymatic activity. In our application, we provided a schematic for the AAV9-hPCCA drug product. In addition, we included descriptive information about the transgene sequence, AAV vector, and the essential elements of the transgene (promoter, intron, *etc.*) which comprise the drug product (Section 4 in Supplementary File S1).

Since we submitted the ODD application in the preclinical development phase, we relied on the inclusion of nonclinical data for demonstrating drug efficacy in a *PCCA*-deficient mouse model of PA.

We provided experimental details, including disease model used, doses administered, and dosing regimen (Section 4.2 in Supplementary File S1). AAV9-hPCCA administration to a *PCCA-*deficient mouse model of PA showed increased survival, restored PCCA protein expression, and improved metabolic parameters, all supportive of the potential benefits that AAV9-hPCCA administration would produce in humans with *PCCA-*related PA. It is important to note that safety or pharmacology/toxicology information would not support scientific rationale and was not included in the ODD request.

While not available in our case, any clinical data available in support of the scientific rationale could be included by a sponsor. Such data can include but are not limited to the study design, treatment population, dosing regimen and ROA, inclusion/exclusion criteria, and outcome measures.^31^ Details of the timing of study drug administration in relation to the onset of the intended disease or condition should be clarified. In addition, if there is an already approved “same drug,” supporting evidence to show clinical superiority of the proposed drug as per FDA criteria is needed to obtain an ODD.^10^ To claim that a new drug is clinically superior and of significant benefit, parameters such as a new formulation or a different ROA will not be sufficient; the sponsor must address safety and efficacy parameters and show major benefit of this drug in patient care.^34^

### Documentation of patient population size: incidence versus prevalence

A population estimate of the proposed disease or condition is quite important for the application and requires that sufficient evidence to support the orphan nature of the disease or condition is provided to facilitate FDA's determination of whether a drug qualifies for ODD.^22^ In instances when a sponsor requests ODD for a drug for only a subset of persons with a particular disease or condition that otherwise affects 200,000 or more people (“orphan subset”), it is required to demonstrate that the remaining persons with such disease or condition would not be appropriate candidates for use of the drug.^22^

FDA OOPD has provided recommendations on their website detailing information required to designate a disease or condition as rare in the United States (Table 2). Notably, the website states: “A Sponsor is expected to make a good faith effort in finding the most recent prevalence data that refers to US population.”^35^ An ODD is generally granted based on prevalence which is defined as the number of persons in the United States who have been diagnosed as having the disease or condition at the time of the submission of the request. For a disease or condition with an acute onset (*i.e.*, <1-year duration), such as rare infections, poisonings, or exposures (*e.g.*, snake bites), incidence estimates can be used. Incidence is defined as the occurrence of new cases of disease or injury in a population over a specified time.

**Table 2.** Food and Drug Administration Office of Orphan Products Development: population size estimate recommendations to support designation of a disease or condition as rare in the United States

*Population size estimate recommendations from FDA OOPD website*
Besides referenced texts and journals, prevalence data for many rare diseases can be found on the internet at government and patient support group websites. Copies of all materials documenting how the prevalence estimate was made should be provided in the designation request. If the reference source is from a website, a hard copy of the document should be included, as well as the website address. The date each website was accessed should also be provided for all website sources referenced.
A sponsor is expected to make a good faith effort in finding the most recent prevalence data that refer to a U.S. population. If only old and/or foreign data are available, the sponsor should explain this in the request. If data are old, the sponsor should explain why the data are still pertinent and, if from a foreign source, why data with that country's population could also be representative of U.S. population.
The sponsor should be reminded that the prevalence estimate must be current to reflect the prevalence at the time of submission of the request for ODD 21CFR §316.21.^38^ To update this estimate, the sponsor should use U.S. population data available from the U.S. Census Bureau.^28^
Provide all calculations and cite references used to make the population estimate.
Estimate should be current at the time of the submission of the ODD request. U.S. Census Bureau data can be used to update any population estimate
When a range of estimates exists, FDA accepts only the largest estimate unless a justification is provided why another estimate is more accurate.
If the drug is intended for diagnosis or prevention of a rare disease or condition, provide the estimated number of people to whom the drug will be administered annually.
If a disease is an acute condition (*i.e*., <1 year duration) incidence may be used as an estimate of the population. However, note that if the disease is a relapsing/remitting disease where each episode is acute in duration, a prevalence estimate may still be required.
If using data from a claims database or foreign data, clearly explain how such data are generalizable to the U.S. population and the limitations of the data.
The National Cancer Institute's SEER Program is one recommended resource for determining cancer statistics in the United States.^39^ A complete prevalence is required.

FDA, Food and Drug Administration; OOPD, Office of Orphan Product Development; SEER, Surveillance, Epidemiology and End Results.

*Source:* FDA OOPD.^35^

**Figure 2.**
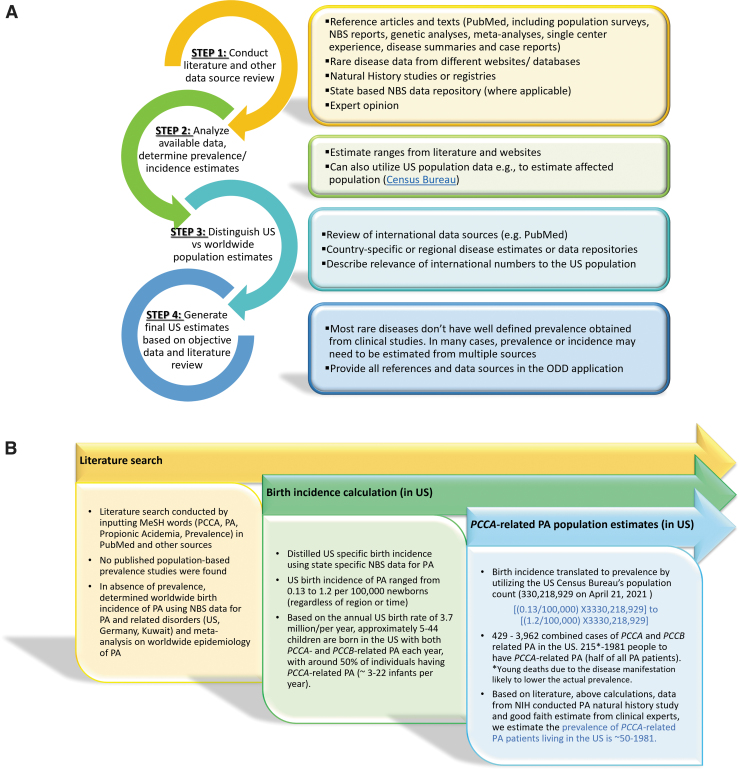
Documentation of population size. **(A)** General search strategy to document current patient population size in the United States; **(B)** steps taken for determining current PA patient population size in the United States. PA, propionic acidemia; NBS, newborn screening; ODD, orphan drug designation.

There are several resources and databases that provide patient population estimates. It is however important to note that some of these may not have the most current information. Therefore it is best to assemble data from several sources to estimate the prevalence/incidence and present the case in the application with appropriate information and references to support it. Shourick et al also provide details of how population estimates can be made using literature quantification.^36^ We have outlined a general search strategy for determining patient population size in the United States (Fig. 2A). Orphanet, a database focused on rare diseases, and Online Mendelian Inheritance in Man (OMIM), a compendium of human genes and phenotypes are also publicly available sources of rare disease information. In the United States, Newborn screening (NBS) is conducted on a state-by-state basis with differences in disease lists for screening, as well as the methods used to screen and identify patients and privacy policies.^37^ Thus, to obtain NBS datasets or information, requests may need to be directed to individual state authorities.

For determining the patient population size for *PCCA-*related PA in the United States, we performed an extensive literature search but did not find reported population-based prevalence studies (Fig. 2B). We did not access NBS databases directly, rather we surveyed literature summarizing data from the NBS studies spanning multiple years in different geographic regions within the United States or outside to understand the worldwide birth incidence of PA.^38–43^ Furthermore, we filtered the published information to establish the birth incidence of PA specifically within the United States. The U.S. incidence data were then translated to estimate the prevalence using census data for the total U.S. population.^44^ In the general population, *PCCA*- and *PCCB*-related forms of PA occur at a similar frequency and, accordingly, we estimated the prevalence of *PCCA*-related PA to be half of all PA patients. We gathered expert opinion from clinicians and NHS^24,25^ to support the final estimated prevalence of *PCCA*-related PA determined to be between 50 and 1,981 patients in the United States (Fig. 2B and Section 8 in Supplementary File S1).

### Additional information

Although not formally requested in FDA regulations, we included in our application package:

(1) a cover letter signed by the sponsor, providing a brief description of the ODD application request, including the investigational drug product and the disease it is intended for;
(2) full reference articles.

### Information specific to Rare Pediatric Drug Designation

We submitted our RPDD request a few months after the ODD approval with much of the same information. The RPDD application needed modification of the request statement and addition of an RPDD justification statement that included the serious nature of the disease, target population, and prevalence in relation to pediatric patients.

## APPLICATION SUBMISSION AND REVIEW

**Figure 3.**
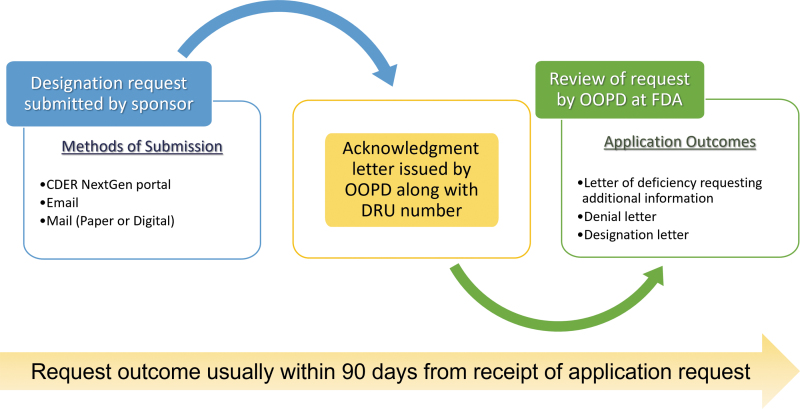
Overview of the submission process. The ODD application can be submitted to the FDA through different submission methods, following which an acknowledgment letter is issued. After review of the application, a decision is made and communicated to the sponsor within 90 days of application receipt, submission, and review.

Once the designation application is ready, the general process for FDA submission and review^10^ is outlined in Fig. 3. After submission, the sponsor (or their agent) receives an acknowledgment letter which includes the OOPD assigned designation request number. Following the FDA OOPD review, a designation letter, a deficiency letter requesting additional information, or a denial letter is issued to the sponsor. The OOPD attempts to review and provide a letter within 90 days from date of application receipt. A RPDD request can be submitted contemporaneously with an ODD application or as a separate proposal. RPDDs submitted at the same time as ODD are reviewed by OOPD within 60 days of submission, whereas RPDDs submitted separately are independently assessed with no set timeline. We submitted the ODD and RPDD applications separately and received our designation letters in 88 and 87 days, respectively.

Please note that as per the ODA, FDA publishes the generic and/or trade name of a drug on their website after ODD is granted.^45^ FDA has provided exhaustive guidance and advice for ODD and RPDD submissions, meetings, content, and communications. Some of these resources are provided in Table 3, and sponsors are urged to consult the FDA OOPD website for the most up-to-date information.^19^

**Table 3.** FDA resources, guidance, and advice for ODD and RPDD applications

Topic	Information Source
General advice	OOPD website: FAQs^35^
How to submit ODD request	OOPD website^19^
Recommended Tips for Creating an Orphan Drug Designation Application	Webinar^32^ script^33^ and slides^31^
Meetings with OOPD	Guidance^52^
ODD Waivers, Reductions, and Refunds	Guidance^12^
FDA OOPD Grants	OOPD website: Orphan Products Grant Program^53^
Rare Pediatric Disease Priority Review Voucher Program	Guidance^17^
Pediatric Subpopulations of Common Diseases	Guidance^54^
Interpreting Sameness of Gene Therapy Products Under the OD Regulations	Guidance^20^

URLs for the websites cited in the table are included in the right column as well as available in the reference list.

FAQ, Frequently Asked Question, OD, Orphan Drug.

## DISCUSSION

The ODD program offers financial incentives for sponsors and has been shown to have a positive correlation with innovation, investor valuation, and investments in rare disease research and development.^46^ There have been 5,099 ODDs and 724 associated drug approvals, comprising both unique and repurposed drugs, since the inception of the program in 1983 until 2019.^47,48^ We also searched the FDA database Orphan Drug Designations and Approvals and found 1,190 designations and 248 approvals from January 2020 until November 2022^49^ suggesting a steady increase in numbers in both categories. Drug approvals span several therapeutic areas and are most concentrated in oncology (34%), followed by metabolic and endocrine (15%) and hematology (11%).^50^ While the proportion of designations for biologics versus small molecules remain stable,^48^ approvals for biologics have almost doubled from 23% in 1980s to 41% in 2010s.^50^ These trends suggest that the ODA has led to important advancements in the number of therapies being developed for treatment, prevention, and diagnosis of rare diseases.

Utilizing keywords such as “adeno-associated,” “AAV,” and “gene therapy,” we also searched for ODDs in the FDA database, specific for AAV gene therapy.^49^ Upon manual curation of the datasets, we have found ∼195 ODDs (Supplementary File S3). Until now, there are three FDA approved AAV gene therapies, Luxturna, Zolgensma, and Hemgenix, for treatments of a rare retinal disorder (RPE65 mutation-associated retinal dystrophy), spinal muscular atrophy, and hemophilia B, respectively.^51^ The observation that there are nearly ten times the number of ODDs for AAV gene therapies compared to approved products reflects a massive expansion in the pipeline for new gene therapies, which is expected to continually grow.

Given the complexity of drug development, coordination among members across different disciplines, including experimentalists, clinical disease experts, and preclinical drug development scientists, has facilitated the rapid preclinical development of an AAV9 gene therapy for PA. We fully acknowledge that programs with less support and expertise will require additional resources, and need more time, to enable an ODD. For these reasons, we plan to share the details of our regulatory documentation to assist those developing therapeutics for rare diseases. We believe that the dissemination activities of the PaVe-GT program such as providing a blueprint of successful ODD and RPDD applications as presented here will reduce the time in drafting these applications and improve their quality, making the process of getting such designations granted more efficient. These efforts will benefit academic and nonprofit drug developers and advance rare disease drug development to improve the health of a large population of patients with unmet medical needs.

## Supplementary Material

Supplemental data

Supplemental data

Supplemental data
