# Supplementary material for: Successfully Navigating Food and Drug Administration Orphan Drug and Rare Pediatric Disease Designations for AAV9-hPCCA Gene Therapy: The National Institutes of Health Platform Vector Gene Therapy Experience
Source: Hum Gene Ther. 2023 Mar 20;34(5-6):217–27. doi: 10.1089/hum.2022.232 (PMC10031144; doi:10.1089/hum.2022.232)
Supplement: Supplemental data [file Supp_FileS1.pdf]

About the AAV9-hPCCA Orphan Drug Designation Request Documents

The following documents are communications between the National Institutes of Health (NIH) National Center for Advancing Translational Sciences (NCATS) and the FDA Office of Orphan Product Development (OOPD) regarding an Orphan Drug Designation (ODD) request to OOPD. The orphan drug is AAV9-hPCCA (NCATS-BL0746) gene therapy for propionic acidemia (PA) resulting from a deficiency of Propionyl-CoA Carboxylase, alpha subunit (PCCA) as part of the Platform Vector for Gene Therapy (PaVe-GT) program.

PaVe-GT is a pilot project that will test whether the efficiency of gene therapy trial startup can be significantly improved by using a standardized process across gene therapies for four different rare diseases. An important goal of PaVe-GT is to share project results and lessons learned with the public in such a way that the information is useful to any party interested in developing a gene therapy efficiently. Specifically, we will make information and results from the PaVe-GT program publicly available in as timely a manner as possible. This includes toxicology and biodistribution data, Investigational New Drug filings and communications with the U.S. Food and Drug Administration, and other study documents. To ensure access to the latest learnings, please visit the PaVe-GT website, subscribe to project updates, and explore the full set of available resources at pave-gt.ncats.nih.gov.

Some portions of this document—primarily sections that are highly specific to *PCCA*-PA and therefore not relevant to other AAV gene therapy efforts—have been edited, redacted or abridged to improve the clarity of materials, and/or support other project objectives. Modified sections are typically identified with italics, brackets, and highlight, *[as shown here]*. The text within the brackets describes the original content.

Disclaimer: NCATS and NIH provides no warranties, representations or guarantees that PaVe-GT Resources will work for any given project or disease condition. Further, NIH disclaims any liability and provides no indemnification. For a full list of terms and conditions for use of PaVe-GT resources, visit pave-gt.ncats.nih.gov.

Table of Contents for PaVe-GT AAV9-hPCCA Orphan Drug Designation Documents

Orphan Drug Designation Request Cover Letter 2

Orphan Drug Designation Request 3

FDA Acknowledgment Letter 28

FDA Response Letter 31

July 01, 2021

Attention: Office of Orphan Products Development, Orphan Drug Designation Program

Re: **Orphan Drug Designation request** for Adeno-Associated Virus 9 human Propionyl-Coenzyme A (CoA) Carboxylase, alpha subunit (AAV9-hPCCA), for the treatment of propionic acidemia (PA) resulting from a deficiency of Propionyl-CoA Carboxylase, alpha subunit (PCCA).

Dear *[Name of Point of Contact within OOPD]:*

Pursuant to 21 CFR 316.20, the National Institutes of Health (NIH), National Center for Advancing Translational Sciences (NCATS), is submitting an Orphan Drug Designation request for Adeno-Associated Virus 9 human Propionyl-CoA Carboxylase, alpha subunit (AAV9-hPCCA) for the treatment of patients with propionic acidemia (PA) resulting from a deficiency of Propionyl-CoA Carboxylase, alpha subunit (PCCA). In addition, we have included a pdf attachment, titled “NCATS AAV9-hPCCA Full References,” consisting of full articles for all referenced citations in our application.

I, *[primary contact name]*, will serve as the primary contact on this submission. *[The names of the alternate contact, regulatory agent, and their respective contact information are listed here]*

For any questions regarding this submission, please contact me at *[the contact information for primary contact]*, including *[the alternate contact and regulatory agent]* listed above.

Sincerely,

[The name, title, and contact information for the primary contact]

National Institutes of Health (NIH), National Center for Advancing Translational Sciences (NCATS)

Adeno-Associated Virus 9 human Propionyl-CoA Carboxylase, alpha subunit (AAV9-hPCCA)

Orphan Drug Designation Request

July 01, 2021

- CONFIDENTIAL –

This document and its contents are the property of and confidential to NCATS. Any unauthorized copying or use of this document is prohibited.

Table of Contents

[The page numbers in the table of contents below have been adjusted from their original values in the request to be consistent with their page numbers in the PaVe-GT AAV9-hPCCA Orphan Drug Designation Documents pdf.]

Section **Page**

LIST OF ABBREVIATIONS ... 6

ORPHAN DRUG DESIGNATION REQUEST STATEMENT ... 7

2 ADMINISTRATIVE INFORMATION ... 8

2.1 Sponsor ... 8

2.2 Primary and Alternate Contacts ... 8

2.3 Regulatory Agent ... 8

2.4 Drug Name ... 8

2.4.1 Chemical Name – Drug Substance ... 8

2.4.2 Generic/Trade Name – Drug Product ... 8

2.5 Manufacturer's Name and Address ... 8

2.5.1 Drug Substance Manufacturer ... 8

2.5.2 Drug Product Manufacturer ... 9

3 DESCRIPTION OF RARE DISEASE OR CONDITION, PROPOSED INDICATION, AND NEED FOR THERAPY ... 9

3.1 Description of Rare Disease or Condition ... 9

3.2 Proposed Indication and Use of AAV9-hPCCA ... 12

3.3 Reasons Why Such Therapy Is Needed ... 12

4 DESCRIPTION OF AAV9-HPCCA AND SCIENTIFIC RATIONALE FOR USE ... 13

4.1 Description of AAV9-hPCCA ... 13

4.1.1 Drug Product Naming Convention ... 13

4.1.2 Drug Product Vector Design ... 13

4.1.3 Drug Product Description, Mechanism of Action and Route of Administration . 14

4.2 Condition Scientific Rationale for the Use of AAV9-hPCCA in the Rare Disease or 14

4.2.1 Clinical Efficacy of AAV9-hPCCA ... 14

4.2.2 Nonclinical Efficacy of AAV9-hPCCA ... 15

5 ORPHAN DRUG STATUS ... 19

6 PATIENT SUBSET CONSIDERATIONS AND MEDICAL PLAUSIBILITY OF THE CHOSEN SUBSET 20
7 REGULATORY STATUS AND MARKETING HISTORY 20
8 DOCUMENTATION OF PATIENT POPULATION SIZE 20
9 REFERENCES 24

LIST OF ABBREVIATIONS

AAV	Adeno-Associated Virus
AAV9	Adeno-Associated Virus serotype 9
AAV9-hPCCA	Adeno-Associated Virus serotype 9 human Propionyl-CoA Carboxylase, alpha subunit
Cas9	CRISPR associated protein 9
CoA	Coenzyme A
CRISPR	Clustered regularly interspaced short palindromic repeats
CRM	Cross Reactive Material
ITR	Inverted Terminal Repeat
LT	Liver Transplantation
2-MC	Total 2-Methylcitrate
MCEE	D-methylmalonyl-CoA epimerase
MMUT	Methylmalonyl-CoA mutase
mRNA	Messenger Ribonucleic Acid
NCATS	National Center for Advancing Translational Sciences
NHGRI	National Human Genome Research Institute
NIH	National Institutes of Health
NHS	Natural History Study
OAA	Oxaloacetic acid
P	Postnatal Day
PA	Propionic Acidemia
PCC	Propionyl-CoA Carboxylase (EC 6.4.1.3)
Pcca	Propionyl-CoA Carboxylase, alpha subunit gene (mouse)
PCCA	Propionyl-CoA Carboxylase, alpha subunit protein
PCCA	Propionyl-CoA Carboxylase, alpha subunit gene (human)
PCCB	Propionyl-CoA Carboxylase, beta subunit gene (human)
POC	Proof of Concept
qPCR	Quantitative Polymerase Chain Reaction
RNA	Ribonucleic acid
ROA	Route of Administration
US	United States
vg	vector genome
WT	Wild Type

ORPHAN DRUG DESIGNATION REQUEST STATEMENT

Pursuant to 21 CFR 316.20, National Institutes of Health (NIH), National Center for Advancing Translational Sciences (NCATS) requests designation of adeno-associated virus serotype 9 human propionyl-CoA carboxylase, alpha subunit (AAV9-hPCCA) as an orphan drug product for treatment of patients with propionic acidemia (PA) resulting from a deficiency of propionyl-CoA carboxylase, alpha subunit (PCCA).

PA is serious and life-threatening disorder caused by deleterious mutations in either the *PCCA* or *PCCB* genes, which encode for the α and β subunits, respectively, of the multimeric enzyme propionyl-CoA carboxylase (PCC). PCC is a ubiquitously expressed enzyme that plays a role in the normal breakdown of branched chain amino acids and odd chain fatty acids. PCC deficiency leads to episodes of metabolic decompensation/acidemia resulting in acute neurological crises, cardiomyopathy, and other serious clinical manifestations. There are currently no approved therapies for the treatment of PA.

PA is a rare disease in the United States (US). There are no population-based studies of the prevalence of PA and almost all studies published in the medical literature relevant to the US population are based on birth incidence calculated from State newborn screening (NBS) data. From these studies, PA has an estimated birth incidence ranging from 0.13 to 1.2 affected infants per 100,000 births.[1,2,3] Based on the annual US birth rate of ~3.7 million/per year in 2019,[4] approximately 5 to 44 children are born in the US with *PCCA*- and *PCCB*-type PA each year, of which 50% of individuals will have *PCCA*-type PA. The most recent study (Adhikari et al, 2020)[1] used both tandem mass spectrometry (MS/MS) NBS data from the State of California (July 2005 through December 2013) and whole exome sequencing (WES), with the results showing a birth incidence of 0.13 PA cases per 100,000 newborns (~6 cases per year), and is likely the most accurate estimate. Based on the US Census Bureau’s population count in 2020 of 330,218,929 people living in the US,[5] the prevalence of PA roughly translates to, at the low end (0.13 cases per 100,000) a prevalence of around 429 to, at the high end, (1.2 cases per 100,000) 3,962 people in the US with PA, half of whom would have *PCCA*-type PA (215-1,981). However, because PA is a life-threatening condition resulting in premature death at a young age for many patients, the true prevalence is likely much lower than this. Based on our good-faith assessment from expert opinion obtained from two of the world’s experts on PA, Oleg A. Shchelochkov, MD and Charles P. Venditti, M.D., Ph.D., NIH, NHGRI, and from data from an NIH-conducted PA natural history study, we estimate there are ~50 *PCCA*-type PA patients living in the US.

Thus overall, the *PCCA*-type PA population potentially amenable to therapy with AAV9-hPCCA gene therapy is a very low prevalence rare disease, with an estimated number of patients ranging from 50 to 3,962 patients in the US, which is far below the 200,000 prevalence cut-off to qualify for an Orphan designation. We therefore request that AAV9-hPCCA be designated as an orphan drug for the treatment of *PCCA*-type PA.

2 ADMINISTRATIVE INFORMATION

2.1 Sponsor

National Institutes of Health (NIH), National Center for Advancing Translational Sciences (NCATS)

9800 Medical Center Dr.
Rockville, MD 20850

2.2 Primary and Alternate Contacts

Primary Contact
[The primary contact and their contact information]

Alternate Contact
[The alternate contact and their contact information]

2.3 Regulatory Agent

[The regulatory agent and their contact information]

2.4 Drug Name

2.4.1 Chemical Name – Drug Substance

Adeno-Associated Virus 9 human Propionyl-CoA Carboxylase, alpha subunit (AAV9-hPCCA)

2.4.2 Generic/Trade Name – Drug Product

AAV9-hPCCA is an Adeno-Associated Virus 9 (AAV9) vector expressing a functional human codon optimized cDNA encoding the Propionyl-CoA Carboxylase, alpha subunit (*PCCA*), under control of *[a specific promoter]*. Details describing the drug product are found in ***Section 4.1***.

[Diagram of the components of the AAV9-hPCCA vector]

2.5 Manufacturer's Name and Address

2.5.1 Drug Substance Manufacturer

[The name, address, and phone number of the drug substance manufacturer]

2.5.2 Drug Product Manufacturer

[The name, address, and phone number of the drug product manufacturer]

3 DESCRIPTION OF RARE DISEASE OR CONDITION, PROPOSED INDICATION, AND NEED FOR THERAPY

3.1 Description of Rare Disease or Condition

Clinical Manifestations

Propionic acidemia (PA) is a rare inborn error of metabolism resulting from deleterious variants in the *PCCA* or *PCCB* genes leading to impaired activity of propionyl-CoA carboxylase (PCC). PCC is a ubiquitously expressed mitochondrial enzyme whose function is closely linked to energy production. It plays a role in the metabolism of the branched chain amino acids valine, methionine, isoleucine and threonine, gut-derived propionate, and odd-chain fatty acids. The clinical course of PA is characterized by chronic multiorgan dysfunction punctuated by episodes of metabolic instability due to metabolic acidosis, ketonuria, hyperammonemia, and hypoglycemia leading to emergency room visits and hospitalizations as seen in ***Figure 1***. Most frequently, PA presents in the neonatal period with hyperammonemia, poor feeding, vomiting, irritability, and lethargy. Without treatment, these infants may develop neonatal encephalopathy, seizures, coma, and respiratory failure, which if left untreated can result in death. Universal newborn screening implemented in the US can identify most affected infants. Infrequently, newborn screening fails to identify affected newborns, usually infants homozygous for the “mild” Amish-Mennonite *PCCB* allele (*PCCB*:c.1606A>G, p.Asn536Asp). Such milder cases can present later in life with life-threatening dilated cardiomyopathy.

Figure 1. Clinical and Laboratory Manifestations of PA

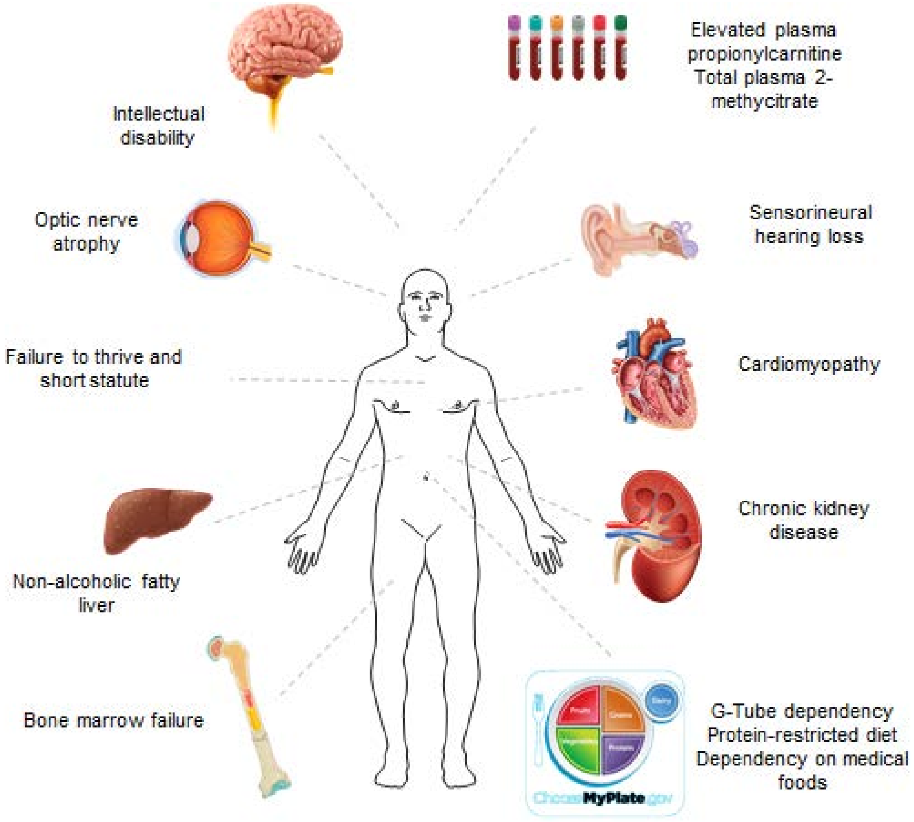

Image source: Shchelochkov, O.A., Manoli, I., Juneau, P. *et al.* Severity modeling of propionic acidemia using clinical and laboratory biomarkers. *Genet Med* **23,** 1534–1542 (2021). https://doi.org/10.1038/s41436-021-01173-2

Propionic acidemia is a rare disorder with birth prevalence varying widely by region. Worldwide, the birth incidence has been reported as high as 1:1000 and as low as 1:500,000. The estimated birth incidence of PA in the United States ranges from 0.13 to 1.2 affected infants per 100,000 births.[1,2,3] (Please see ***Section 8*** for detailed discussion on birth incidence).

The long-term prognosis for survival in severely affected patients is poor, which was first illustrated in the 1990s by an early and relatively large (for the disease prevalence) single center study of 20 patients with PA treated at a tertiary care center.[6] This study showed that patients who presented in the first week of life (11 patients) largely perished by the age of 6 years. Even with treatment, patients who survived suffered from recurrent metabolic instability often precipitated by periods of metabolic stress, such as infections, and can develop chronic progressive multisystemic complications, including cardiomyopathy. Over the subsequent decades, it has been recurrently noted that PA patients with an early and severe clinical course experience increased mortality and disease associated morbidity.[7] The recalcitrant nature of the disorder to conventional medical management, including the dietary restriction of amino acid

precursors, L-carnitine supplementation, and administration of oral metronidazole to reduce the generation of propionic acid by intestinal bacteria, has led to the implementation of elective liver transplantation (LT) as an experimental surgical treatment for PA. While not curative of all aspects of disorder, successful LT in the setting of PA may improve metabolic stability and protection from early death, and therefore represents a clinical benchmark for gene replacement or other approaches that might increase hepatic PCC expression and activity.

Pathophysiology

PCC is a ubiquitously expressed, heteropolymeric mitochondrial enzyme involved primarily in the catabolism of propionogenic amino acids, particularly isoleucine, valine, methionine, and threonine, as well as odd-chain fatty acids. The enzyme is composed of α- and β-subunits encoded by their respective genes, *PCCA* and *PCCB*, and PA is caused by pathogenic variants in either gene. PCC catalyzes the first step in the conversion of propionyl-CoA to D-methylmalonyl-CoA in the pathway of propionyl-CoA oxidation, depicted in ***Figure 2***. The formation of 2-methylcitrate (2-MC), an important biomarker likely generated through the condensation of oxaloacetic acid (OAA) and propionyl-CoA is also noted, as are downstream enzymatic steps in the pathway, including D-methylmalonyl-CoA epimerase (MCEE) and methylmalonyl-CoA mutase (MMUT), in the metabolism of propionyl-CoA into the citric acid (Krebs) cycle.

Figure 2. Catabolism of Propionyl-CoA.

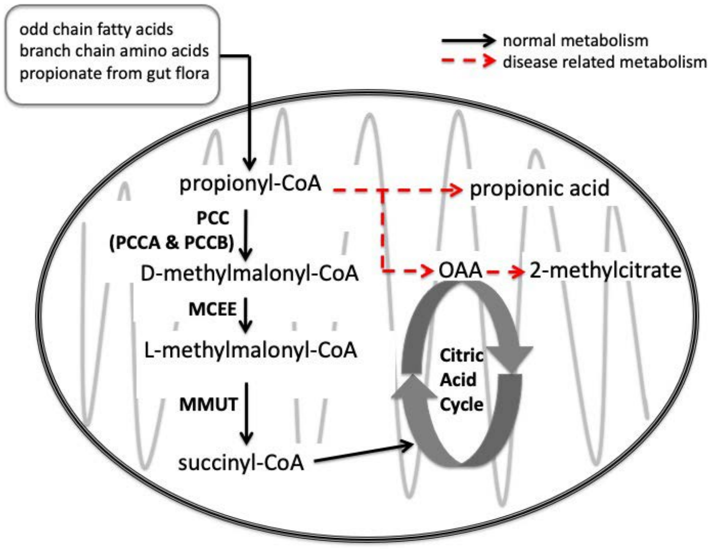

3.2 Proposed Indication and Use of AAV9-hPCCA

The proposed clinical use of AAV9-hPCCA is for the treatment of patients with PA resulting from a deficiency of PCC due to deleterious variants in PCCA. Briefly, AAV9-hPCCA is an AAV9 vector expressing a functional human codon optimized cDNA encoding PCCA, under control of *[a specific promoter]*. In a newly generated knockout murine model, designated *Pcca*$^{-/-}$, AAV9-hPCCA demonstrated efficacy. The *Pcca*$^{-/-}$ animals have no detectable Pcca protein and display immediate neonatal lethality, akin to the phenotype of the human disorder. Upon retro-orbital injection of AAV9-hPCCA in these animals, *[description of dose responses]* was observed. In an initial biodistribution study of AAV9-hPCCA treated *Pcca*$^{-/-}$, *PCCA* mRNA and PCCA protein were found in both target tissues, heart and liver, as analyzed by qPCR and Western blotting, respectively. The *Pcca*$^{-/-}$ animals also display higher levels of the 2-MC biomarker in the plasma which was substantially reduced upon administration of AAV9-hPCCA.

3.3 Reasons Why Such Therapy Is Needed

There are currently no approved therapies for PA and novel therapies are urgently needed to prevent irreversible disease complications and improve patient management. PA manifests life-threatening debilitating illness in early childhood and is poorly responsive to available medical therapy. Adult PA patients have high prevalence of severe kidney and heart disease, and irreversible disease complications to sensory organs. Current standards of care include strict adherence to low-protein diet with and without poorly palatable medical foods, and supplementation with levocarnitine. Other treatments may apply if patients develop severe complications of PA, such as, the use of cardiac medications in patients with cardiomyopathy or antiepileptic drugs in PA patients with epilepsy.[7] The long-term prognosis for survival in severely affected patients is poor as illustrated by an early and relatively large (for the disease prevalence) single center study of 20 patients with PA treated at a tertiary care center: Those who presented in the first week of life (11 patients) died by the age of 6 years.[6] Over the decades, it has been recurrently noted that PA patients with an early and severe clinical course experience increased mortality and disease associated morbidity.[7]

Some PA patients, who experience frequent hospitalizations due to metabolic instability and/or who develop dilated cardiomyopathy may undergo liver transplantation (LT) and require life-long immunosuppression. While not curative of all aspects of the disorder, successful LT in the setting of PA provides restoration of metabolic stability and protection from early death, and therefore represents a clinical benchmark for gene replacement or addition approaches that might increase hepatic PCC expression and activity. Although the post-LT status in PA remains to be fully elucidated, some patients have developed renal disease and even cardiomyopathy after successful LT. An additional and potentially fatal complication,

presumed secondary to chronic exposure to immune suppression, has been the development of post-transplant lymphoproliferative disease. Therefore, while LT substantially improves the clinical and metabolic phenotypes seen in severe PA patients, it clearly carries immediate/acute (post-surgical) and chronic/subacute risks. In theory, these risks could be mitigated or perhaps eliminated by successful liver-directed gene therapy with AAV9-hPCCA, which we propose as a single IV infusion aiming to restore therapeutic levels of PCCA in the liver and heart.

4 DESCRIPTION OF AAV9-HPCCA AND SCIENTIFIC RATIONALE FOR USE

4.1 Description of AAV9-hPCCA

4.1.1 Drug Product Naming Convention

Adeno-Associated Virus 9 human Propionyl-CoA Carboxylase, alpha subunit (AAV9-hPCCA)

4.1.2 Drug Product Vector Design

AAV9-hPCCA is an AAV9 vector expressing a functional human codon optimized cDNA encoding *PCCA*, under control of *[a specific promoter]*. In humans, endogenous PCCA protein is ubiquitously expressed, therefore we designed a therapeutic transgene cassette with a constitutive promoter to enable wide expression and selected the AAV9 capsid to further enable hepatic and cardiac transduction. A schematic of the vector transgene, description of cassette features summarizing the salient features of the AAV vector and the vector map is presented in ***Figure 3*** and ***Figure 4***. The complete nucleotide sequence of AAV9-hPCCA is included as ***Appendix A.***

Figure 3. A Schematic of the Vector Transgene, Description of the Cassette.

[Diagram of the components of the AAV9-hPCCA vector, previously included under 2.4.2]

[Table describing basic elements of the cassette including: the length of the inverted terminal repeats, whether there is an enhancer, what is the promoter, if there is an intron, if there is a 5' untranslated sequence, the nature of the therapeutic gene (e.g. natural or codon-adjusted), if there is a 3' untranslated sequence included, the polyadenylation signal, 3' inverted terminal repeat, comments about genome configuration (e.g. single strand or double strand) and additional remarks regarding the regulatory sequences and/or transgene, such as microRNA binding sites.]

Figure 4. A Schematic of the Vector Transgene, Description of the Vector Map.

[A map of the plasmid used to produce the therapeutic transgene. In addition to noting major components of the therapeutic transgene, other features such as antibiotic resistance, origin of replication, and salient details surrounding the plasmid backbone were included. The size of the transgene and constitutive elements were denoted.]

4.1.3 Drug Product Description, Mechanism of Action and Route of Administration

AAV9-hPCCA is a biologic modality for introducing corrected DNA into PA patients with biallelic pathogenic variants in the *PCCA* gene. The gene is delivered using an AAV virus, serotype AAV9, a modified virus that is not infectious in humans but maintains its natural ability to deliver genetic material into cells. The AAV9-hPCCA will introduce a normal copy of the human *PCCA* gene and help restore the normal function of the PCC enzyme in the catabolism of propionyl-CoA, as described in ***Figure 2***, ***Section 3.1***. AAV9-hPCCA will be stored at -80°C and delivered in patients as an intravenous (IV) solution using the peripheral IV route of administration (ROA). The final formulation for the drug product has not been determined but the formulation for an initial batch for preclinical *in vivo* animal studies was *[formulation details]*.

4.2 Scientific Rationale for the Use of AAV9-hPCCA in the Rare Disease or Condition

PA is caused by biallelic pathogenic variants in either the *PCCA* or *PCCB* gene. It is caused by a deficiency of PCC, a ubiquitously expressed, heteropolymeric mitochondrial enzyme involved primarily in the catabolism of propionogenic amino acids. The enzyme is composed of α- and β-subunits encoded by their respective genes, *PCCA* and *PCCB*. PCC catalyzes the first step in the conversion of propionyl-CoA to D-methylmalonyl-CoA in the pathway of propionyl-CoA oxidation, depicted in ***Figure 2***, ***Section 3.1***. Replacement of the PCCA enzyme through delivery of AAV9-hPCCA gene therapy is expected to help normalize the metabolic defects associated with PA.

4.2.1 Clinical Efficacy of AAV9-hPCCA

To date, no clinical data have been collected with AAV9-hPCCA for the treatment of patients with PA resulting from a deficiency of PCCA protein. However, as stated previously, PA patients that have undergone successful LT demonstrate a restoration of metabolic stability and protection from early death, and therefore represents a clinical benchmark for gene replacement therapy that may lead to increased hepatic PCC expression and activity.

There is a lack of natural history longitudinally evaluating PA biomarkers and their association

with outcomes corresponding to survival, functioning and disease burden. To close this gap, the Organic Acidemia Section of National Human Genome Research Institute (NHGRI) initiated a Natural History Study (NHS) of the PA population (National Clinical Trial # NCT02890342)[8] The study is being conducted at the NIH Clinical Center and has enrolled 40 participants ages 2 years and older (2.5 years – 54 years of age). The cohort features a genetically heterogeneous PA population, a high proportion of adult patients (~35%), and a subset of liver- and kidney-transplanted PA participants. A recent publication has identified candidate biomarkers in this cohort.[8]

4.2.2 Nonclinical Efficacy of AAV9-hPCCA

***In Vitro* Studies in Human Liver (*[cell line name]*) PCCA Knockout (KO) Cell Line**
Infection of an engineered human liver *[cell line name]*/PCCA KO cell line incubated with varying concentrations (*[list of concentration values]*) of AAV9-hPCCA showed *[pattern of expression]* of PCC enzyme by that was not seen in the control (uninfected KO cells) (***Figure 5*****)**. In addition, cell lysates from *[cell line name]*/PCCA KO infected cells also showed *[description of dose response]*in PCC enzyme activity as measured by a *[method]* that quantitates the conversion of propionyl-CoA to methylmalonyl-CoA (not presented). The *[pattern of]* PCC protein expression observed after AAV9-hPCCA infection is consistent with the *in vivo* *[pattern of]* expression of PCC protein measured in liver extracts from *Pcca*$^{-/-}$ mice after retroorbital injection of AAV9-hPCCA (***Figure 8***).

Figure 5. AAV9-hPCCA *In Vitro* Infection Studies in Human Liver (*[cell line name]*) PCCA Knockout (KO) Cell Line

[Image: Western blot results]

A deletion mutation in *PCCA* was engineered in a human hepatocyte derived cell line (*[cell line name]*) to create the PCCA KO cell line, and used to test the in vitro potency of AAV9-hPCCA applied at varying moiety of infections ranging from *[lower concentration value]* to *[higher concentration value]*. After 48 hours, the infected PCCA KO cells were harvested and lysed, and *[a quantity]* of total cellular protein was subjected to Western analysis using a PCCA polyclonal antibody. Beta-actin was used as a loading control. *[Description of dose response.]*.

***In Vivo* Studies in a Lethal Mouse Model of PA Used to Investigate Effect of AAV9-hPCCA**
Gene therapy has been administered to different animal models of PA caused by PCCA deficiency and reduction in metabolite levels indicated therapeutic potential of gene therapy.[9, 10, 11, 12] However, the previous models of PA were made by creating large genetic deletions in the *Pcca* gene or relied on transgenic constructions and did not fully recapitulate the genetic mutations or disease phenotype commonly observed in humans. Hence, for testing of AAV gene therapy vectors for human translation, we generated mice using CRISPR/Cas9 genome editing to

engineer mutations that are compatible with those seen in the patients, such as frameshift-stop and missense changes. The mutation, Pcca$^{[mutation\ name]}$, caused by a deletion in the *Pcca* gene, is severe and null at the level of PCCA protein expression (cross reactive material, CRM-). Mice homozygous for this mutation (*Pcca*$^{-/-}$) were generated and recapitulate the neonatal lethal form of PA in humans. These mice perish in the immediate neonatal period (within *[period of time]* of birth) without gene therapy treatment, and hence, are particularly useful to assay the efficacy of AAV9-hPCCA gene therapy for the treatment of PA. Data from the *in vivo* studies demonstrate that administration of AAV9-hPCCA vector rescues *Pcca*$^{-/-}$ neonatal mice by increasing animal survival and reducing levels of the pathological metabolite.

We have conducted a series of studies in a newly generated knockout mouse model, designated *Pcca*$^{-/-}$ and detailed below. The mice have no detectable PCCA protein and display immediate neonatal lethality, akin to the phenotype of the human disorder. A summary of the POC studies conducted in *Pcca*$^{-/-}$ neonatal mice after administration of AAV9-hPCCA thus far is presented below in ***Table 1***. The gene therapy shows a pronounced effect on the animal survival and reduction in the plasma levels of the pathological metabolite, 2-MC.

Table 1. Survival and Metabolic Phenotype Summary of *Pcca*$^{-/-}$ Animals Treated with Different Doses of AAV9-hPCCA

Treatment Group	Dose (vg/kg)	No. of *Pcca*$^{-/-}$ Mice	Mean Survival (Days) ± Standard Deviation	Metabolic Phenotype (2-MC)
Untreated	N/A	*[value]*	*[value]*	*[value]*
[Vehicle]	N/A	*[value]*	*[value]*	*[value]*
*AAV9-hPCCA	*[lower dose]*	*[value]*	*[value]*	*[value]*
*AAV9-hPCCA	*[mid dose]*	*[value]*	*[value]*	*[value]*
*AAV9-hPCCA	*[higher dose]*	*[value]*	*[value]*	*[value]*

***Note:** Different lots of research grade AAV9-hPCCA were used for the *Pcca*$^{-/-}$ studies listed in the table above, but all contained the same therapeutic transgene, and all animals were dosed by retro-orbital injection on post-natal day P0-1.**

Detailed Experimental Design and Results

For definitive testing of AAV gene therapy vectors for human translation, we created mice using CRISPR/Cas9 genome editing to engineer mutations that are compatible with those seen in the patients, such as frameshift-stop and missense changes. One mutation, Pcca$^{[mutation\ name]}$ in Exon *[value]* of the *Pcca* gene is severe and null at the level of protein expression (CRM -). Please see ***Table 2***.

Table 2. ***Pcca***$^{-/-}$ **Neonatal Knockout Mouse Model**

Pcca exon Targeted	Pcca Protein Mutation	Homozygous phenotype
[value]	*[mutation name]*	Neonatal lethal CRM negative<br>Increased 2-MC

Building on many years of previous work with AAV gene therapy in related mouse models of methylmalonic acidemia, where early lethality is a uniform characteristic of the homozygous mutant phenotype, we initiated our studies to determine the therapeutic efficacy of AAV9-hPCCA delivered by retro-orbital plexus injection in newborn *Pcca*$^{-/-}$ mice. Because the untreated *Pcca*$^{-/-}$ mice experience *[percentage value]* lethality by P2 (***Figure 6***), we injected AAV9-hPCCA via retro-orbital plexus to the systemic circulation, to recapitulate IV delivery, the anticipated ROA in humans.

Survival of AAV9-hPCCA Treated ***Pcca***$^{-/-}$ **Mice**

Homozygous *Pcca*$^{-/-}$ males rescued by AAV9 gene delivery were mated with heterozygous females. All the pups obtained in the litter were treated with AAV9-hPCCA within a few hours after birth (at P0-1). For the survival study in the *Pcca*$^{-/-}$ mice, we tested three doses of AAV9-hPCCA, *[lower dose]* vg/kg (n=*[value]*), *[mid dose]* vg/kg (n=*[value]*) and *[higher dose]*vg/kg (n=*[value]*), administered via retro-orbital injections. To minimize stress on the newborns and mothers, the pups were not weighed before AAV9-hPCCA administration, the vector was dosed as vg/pup and approximated for vg/kg using the average body weight of *[weight in grams]* (n=*[value]*). The genotypes of all pups were not determined until after injection.

Table 3 below summarizes the doses administered to the homozygous *Pcca*$^{-/-}$ neonatal animals. Subsequently, survival was monitored, and the transgene expression was measured. At selected timepoints, biomarker responses (2-MC) and vector genome biodistribution were measured.

Table 3. List of AAV9-hPCCA Treated Neonatal ***Pcca***$^{-/-}$ **Mice.**

Genotype	Treatment	Dose (vg/pup)	Dose (vg/kg)	Sex	Number of Mice
Pcca$^{-/-}$	None	N/A	N/A	Not Available	*[value]*
Pcca$^{-/-}$	*[Vehicle]*	N/A	N/A	Not Available	*[value]*
Pcca$^{-/-}$	AAV9-hPCCA	*[value]*	*[lower dose]*	M	*[value]*
Pcca$^{-/-}$	AAV9-hPCCA	*[value]*	*[lower dose]*	F	*[value]*
Pcca$^{-/-}$	AAV9-hPCCA	*[value]*	*[lower dose]*	Not Available	*[value]*
Pcca$^{-/-}$	AAV9-hPCCA	*[value]*	*[mid dose]*	M	*[value]*
Pcca$^{-/-}$	AAV9-hPCCA	*[value]*	*[mid dose]*	F	*[value]*
Pcca$^{-/-}$	AAV9-hPCCA	*[value]*	*[mid dose]*	Not Available	*[value]*
Pcca$^{-/-}$	AAV9-hPCCA	*[value]*	*[higher dose]*	M	*[value]*
Pcca$^{-/-}$	AAV9-hPCCA	*[value]*	*[higher dose]*	F	*[value]*

The results for the various dose cohorts of AAV9-hPCCA treated *Pcca* $^{-/-}$ mice are presented in ***Figure 6***. *[Description of dose(s) that provide rescue from neonatal lethality, and prolong the life of the mutant mice.]* The control *Pcca* $^{-/-}$ animals (untreated/*[vehicle]* treated) died shortly after birth. The animals dosed at *[lower dose]* had a mean survival of *[value]* days, those in the *[mid dose]* cohorts survived for *[value]* days, and animals in the highest dose group of *[higher dose]* had a mean survival of *[value]*.

Figure 6. Survival Curve of AAV9-hPCCA Treated *Pcca*$^{-/-}$ Mice.

[Graph of survival curve. The y axis of the graph is percent survival of PCCA-/- mice, from 0 to 100. The x axis is number of days. Each line represents a different experimental group/dose.]

Mice were treated with indicated doses of AAV9-hPCCA at P0-1. *[Vehicle]* and no treatment (untreated) were used as a control. The graph depicts the percent survival of different cohorts of animals ** *P*<0.0001, ** *P*<0.01 compared to survival of untreated vs treated *Pcca*$^{-/-}$ mice. *P* values were calculated using a Log_rank (Mantel-Cox) test.**

Measurement of Plasma 2-MC in AAV9-hPCCA Treated *Pcca*$^{-/-}$ Mice

Animals dosed with AAV9-hPCCA were bled to measure a biomarker response (at *[multiple timepoints]*), and then sacrificed to assess transgene expression and vector biodistribution. On *[early timepoint]*, the weights of the AAV9-hPCCA treated *Pcca*$^{-/-}$ mice were not different than treated control littermates (not presented). As illustrated in ***Figure 7***, treatment of *Pcca*$^{-/-}$ mice with AAV9-hPCCA resulted in a substantial reduction in the plasma levels of 2-MC as compared to the untreated animals at doses of *[list of applicable doses]*.

Figure 7. Plasma 2-MC Levels in AAV9-hPCCA Treated *Pcca*$^{-/-}$ Mice.

[Graph of Plasma 2-MC levels for untreated mice and treated mice at the lower, mid and higher dose]

Mean $\pm$ S.D. of the plasma 2-MC levels in *Pcca*$^{-/-}$ mice treated at birth with AAV9-hPCCA. **P*<0.01 for *Pcca*$^{-/-}$ treated vs untreated at the time of birth calculated by a two sided, two tailed t-test.

Transgene Expression in AAV9-hPCCA Treated *Pcca*$^{-/-}$ Mice

After treatment with AAV9-hPCCA, the transgene and protein levels were assessed in the liver of a small number of *Pcca*$^{-/-}$ mice treated with *[two of the dose levels]* of AAV9-hPCCA. The *PCCA* mRNA was quantified by qPCR and the PCCA protein levels were measured by Western blotting at *[two timepoints]*. The mRNA levels were normalized to endogenous glyceraldehyde 3-phosphate dehydrogenase (*Gapdh*) expression and protein levels were normalized to endogenous beta actin levels. The AAV encoded *PCCA* mRNA varied between *[range of*

percentage values] of WT *Pcca* levels and the protein expression was at ~ *[value]*% of the wild-type murine PCCA level (***Figure 8***).

Figure 8. Hepatic *PCCA* mRNA and PCCA Protein Expression in AAV9-hPCCA Treated *Pcca*$^{-/-}$ Mice

[A clustered bar graph reporting percent of WT PCCA expression and relative PCCA mRNA expression across several experimental groups: WT; untreated Pcca $^{-/-}$; and treated Pcca $^{-/-}$ mice at specific doses and timepoints]

Quantitation (Mean ± S.D.) of *PCCA* mRNA and PCCA protein represented as a percentage of endogenous WT expression levels and normalized to beta-actin for protein levels and to *Gapdh* for mRNA expression.

Biodistribution in AAV9-hPCCA Treated Mice

In the PA patients, the target tissues are the liver and heart, and therefore, our initial survey was focused on hepatic and cardiac studies in mice treated with the AAV9-hPCCA vector. Using *[method]* to detect the *PCCA* transgene and the endogenous *Gapdh* locus, the copy numbers of the *PCCA* transgene were assessed at *[timepoints]* in heterozygous *Pcca*$^{+/-}$ and homozygous *Pcca* $^{-/-}$ animals dosed with AAV9-hPCCA at *[doses]*. As can be seen in ***Figure 9***, the AAV9-hPCCA genome is readily detected, indicating *[transduction results]*. Furthermore, the relative transduction between *Pcca*$^{-/-}$ mice and *Pcca*$^{+/-}$ *[is described in Figure 9]*.

Figure 9. Biodistribution of AAV9-hPCCA in Liver and Heart Tissues

[A cluster bar graph, providing the vector copy number of the PCCA transgene normalized to alleles of the Gapdh gene, as found in the liver and heart in Pcca-/- and Pcca+/- mice at different timepoints and doses.]

Vector copy number of the *PCCA* transgene in the liver and heart normalized to alleles of the *Gapdh* gene using *[method]* in the livers and hearts of AAV9-hPCCA treated *Pcca*$^{-/-}$ and *Pcca*$^{+/-}$ mice at *[timepoint]* and *[later timepoint]* post-treatment. Error bars are ± S.D.

5 ORPHAN DRUG STATUS

AAV9-hPCCA is not designated as an orphan drug or product for the treatment of patients with PA resulting from a deficiency of PCCA, and AAV9-hPCCA is currently not designated as an orphan drug or product in the United States for any other indication.

6 PATIENT SUBSET CONSIDERATIONS AND MEDICAL PLAUSIBILITY OF THE CHOSEN SUBSET

The prevalence of PA does not exceed 200,000. Therefore, subset considerations and medical plausibility of a subset are not applicable to the proposed indication of patients with PA resulting from a deficiency of PCCA in this request.

7 REGULATORY STATUS AND MARKETING HISTORY

NCATS is currently in late phase pre-clinical development of AAV9-hPCCA, where we have an Initial Targeted Engagement for Regulatory Advice on CBER Products (INTERACT) meeting (submission #*[unique identifier]*) scheduled with FDA, Center for Biologics Evaluation and Research (CBER), Office of Tissues and Advanced Therapies (OTAT), on July 14, 2021, to discuss our completed proof of concept (POC) studies and planned IND enabling toxicology studies in preparation for IND submission fourth quarter of 2022.

AAV9-hPCCA is not currently marketed or approved for use in any country, including the United States. In accordance with section 526(a)(1) of the Federal Food, Drug, and Cosmetic Act [21 USC 360bb(a)(1)]; 21 CFR 316.20(b)(7); and 21 CFR 316.23(a), NCATS has not previously submitted a marketing application to FDA for the same active moiety in AAV9-hPCCA for the same rare disease or condition prior to submission of the AAV9-hPCCA orphan drug designation request.

8 DOCUMENTATION OF PATIENT POPULATION SIZE

PA is a rare disease in the United States (US), which includes PCC enzyme deficiency from deleterious mutations in either the *PCCA* or *PCCB* genes. The *PCCA*- and *PCCB*-types appear to occur with equal distribution and have similar disease severity and manifestations.[7] There are no population-based prevalence studies, and most of the data used to estimate prevalence are from birth incidence mainly from studies conducted on newborn screening (NBS) data identified in the medical literature. Overall, from these sources, PA in the US is a very low incidence/prevalence rare disorder, with birth incidence ranging from approximately 0.13-1.2 per 100,000 in the US, regardless of region or time period examined. Based on the annual US birth rate of 3.7 million/per year,[4] approximately 5-44 children are born in the US with both *PCCA*- and *PCCB*-type PA each year, with around 50% of individuals having *PCCA*-type PA (approximately 3-22 infants per year).

Studies identified from the medical literature are summarized as follows:

- The most recent study was performed and published by Adhikari et al in 2020,[1] which reported the experience of the NBSeq project that evaluated whole-exome sequencing (WES) as a new methodology for NBS. In this study, the authors obtained archived residual dried

blood spots (DBS) and data for nearly all inborn errors of metabolism (IEM) cases (n=1,728 DBSs) identified using tandem mass spectrometry (MS/MS) from 4.5 million screened infants born in California between July 2005 and December 2013. They then compared the MS/MS with the results from WES. This was the largest study to date on sequencing efforts of an entire population of IEM-affected cases identified from NBS, inclusive of 48 different IEMs and the 78 genes associated with these disorders, representing 1,190 newborns: 805 IEM-affected newborns and 385 MS/MS false positives. There were 237 affected individuals with an organic acid disorder, of which 6 newborns were shown to have been diagnosed with PA, which approximates to a birth incidence of 0.13 per 100,000.

- Chapman et al, 2018,[2] conducted a study on NBS results for 3 disorders, maple syrup urine disease, PA, and methylmalonic aciduria (MMA), from 3 geographic regions around the world, including state screening labs in the US, the southwest region of Germany, and Kuwait. The US data was collected and analyzed from the US NBS data base from 1991 to 2000, and positive results from the diseases in questions were compared to the on-line reported birth rates per US states. Some states reported MMA and PA together, given the marker C3 being common to both. For PA alone, there were a total of 12 cases identified from a total of 2,912,901, for a birth incidence of 1:242,741, or 0.41 cases per 100,000. For MMA and PA together, there were 147 cases in 7,544,243 births, for a birth incidence of 1:50,709 (MMA alone birth incidence was 1:69,354, 3.5 times the incidence of PA alone).
- Almasi et al, 2019,[3] conducted a systematic literature review and meta-analysis on the worldwide epidemiology of PA, which estimated point prevalence of PA per 100,000 births calculated separately by region (North American, Europe, Asia-Pacific, Middle-East and North Africa (MENA)), and in 2 time periods 1981-2000, and 2001-present. They identified 43 studies included in the qualitative synthesis on epidemiology, and 31 studies included in the quantitative synthesis. The vast majority of articles reported on newborn screening programs (NBS) providing estimates on the birth prevalence of the disease, defined as the number of affected newborns divided by the total population screened, 8 of which described results from NBS programs in the US and are summarized in ***Table 4***. There were 6 studies used for the North America (NA) point estimate. The results showed that for NA, birth prevalence ranged from 0.20 (California, US) to 1.35 (Ontario, Canada) per 100,000 newborns, with the pooled point estimate of 0.33 per 100,000 newborns in NA. Similar rates were noted in Europe, and higher rates in Japan and MENA. Pooled point estimates remained below 1 per 100,000 newborns in all regions except MENA, which were significantly higher (>3 per 100,000). The authors additionally noted a scarcity of studies in PCCA- and PCCB-subtypes but are reported to be approximately equally distributed,[7] and that broadly targeted population-based prevalence studies are not available. Their overall conclusions were that OA is an "ultra-rare" disorder; however, ultra-rare was not specifically defined.
    - Of note, PA prevalence by ethnicity in the US was investigated in only one study, Feuchtbaum et al (2012),[13] who noted that only Native Americans were characterized by

a significantly higher detection rate (6.7 per 100,000 newborns) than the overall rate (0.2 per 100,000) in the US.

Table 4: Brief Summaries of the US Studies Included in the Almasi Meta-Analysis[3]

Lead Author, Year of Publication	Estimated Birth Incidence per 100,000 Newborns	Brief Description
Feuchtbaum, 2012[13]	0.2	Study to describe birth prevalence of genetic disorders among different racial/ethnic groups through analysis of population-based NBS data from 2005-2010, inclusive of 2,282,138 newborns screened. Calculated birth incidence 0.2 per 100,000 newborns screened.
Frazier, 2006[14]	0.33	Report of North Carolina's 8-year experience with MS/MS NBS, and incidence of disease for selected amino acid, fatty acid and organic acid disorders, 1997-2005. Calculated incidence 1:300,000
Therrell, 2014[15]	0.42	Tabulation of 10-year NBS data for selected IEM from 2001-2011 from the National Newborn Screening Information System (NNSIS). Identified 105 PA cases in 25,026,374 newborns screened, for a birth incidence of 1:238,346 (0.42 per 100,000)
Weisfeld-Adams, 2009[16]	0.5	Retrospective DBS data analysis in patients with molecularly confirmed cobalamin C (cblcC) disease, in 4-year period (2005-2008) in NY. Total number of infants screened = 1,006,325, 5 cases of PA confirmed (1:201,265).
Comeau, 2004[17]	0.6	Study summarizes the New England Newborn Screening Program's (NENBSP) approach and experience, from Jan 1999- Jan 2003. PA clustered into panel testing, and not separately broken out within the paper. Birth incidence estimated to be around 0.6 per 100,000.

Lead Author, Year of Publication	Estimated Birth Incidence per 100,000 Newborns	Brief Description
Naylor, 1999[18]	0.71	Study reporting results of NBS using MS/MS of more than 700,000 newborns from Pennsylvania, Ohio, North Carolina and Louisiana, which detected 163 IEMs. 32 patients were identified with organic acidemias, of which 5 had PA (0.71 per 100,000).
Chace, 2001[19]	~0.7	Study report on the validation of MS/MS analysis for MMA and PA from DBS obtained from 908,543 newborns from NBS from approximately 1992-2001. Results showed 1:64,896 with either MMA or PA by MS/MS, but birth incidence was not broken out by MMA or PA individually.
Zytkovicz, 2001[20]	1.2	Study summarizes 2-year MS/MS results for amino acid and fatty and organic acid disorders from DBS from the NENSP from Feb 1999 to Feb 2001, inclusive of 164,000 newborns. 36 infants were identified for C3, of which 2 were PA patients, for an estimated birth incidence of 1.2 per 100,000.

Thus overall, these findings are notable for the following:

First, there are no population-based prevalence studies for PA for the US population in the medical literature.

Second, all of the studies identified were based on birth incidence calculated from state NBS results in various regions around the US and in different time periods, mainly after 1999 when screening based on C3 levels detected by MS/MS became more broadly available. Birth incidence ranged from 0.13 to 1.2 per 100,000 newborns, and the results were largely similar across the US and over time. This translates to about 5-44 children born with PA per year, with 50% of these expected to be *PCCA*-type PA (3-22 cases per year). The most recent analysis conducted by Adhikari et al, 2020,[1] compared MS/MS with WES, which likely addressed false positives and is likely the most accurate. This study showed a birth incidence of 0.13 cases of PA per 100,000 newborns (6 cases per year), half of which are expected to be *PCCA*-type PA.

Third, based on the US Census Bureau's population count in 2020, there were 330,218,929 people living in the US.[5] This roughly translates to a PA prevalence ranging from, at the low end

(0.13 cases per 100,000) of around 429 people in the US having PA to, at the high end, (1.2 cases per 100,000) of 3,962 people with PA, half of whom would have *PCCA*-type PA (215-1,981). However, because PA is a life-threatening condition resulting in premature death at a young age for many patients, the true prevalence is likely lower than this. Based on our good-faith assessment from expert opinion obtained from two of the world's experts on PA (Oleg A. Shchelochkov, MD and Charles P. Venditti, M.D., Ph.D., NIH, NHGRI), and from data from an NIH-conducted PA natural history study,[8] we estimate there are ~50 *PCCA*-type PA patients living in the US.

In conclusion, the *PCCA*-type PA population amenable to therapy with AAV9-hPCCA gene therapy is a very low incidence/prevalence rare disease, with an estimated number of patients ranging from 50-3,962 patients in the US, which is far below the 200,000 prevalence cut-off to qualify for an Orphan designation. We therefore request that AAV9-hPCCA be designated as an orphan drug for the treatment of *PCCA*-type PA.

9 REFERENCES

1. Adhikari AN, Gallagher RC, Wang Y, Currier RJ, Amatuni G, Bassaganyas L, Chen F, Kundu K, Kvale M, Mooney SD, Nussbaum RL, Randi SS, Sanford J, Shieh JT, Srinivasan R, Sunderam U, Tang H, Vaka D, Zou Y, Koenig BA, Kwok PY, Risch N, Puck JM, Brenner SE. The role of exome sequencing in newborn screening for inborn errors of metabolism. Nat Med. 2020 Sep;26(9):1392-1397. doi: 10.1038/s41591-020-0966-5.

2. Chapman KA, Gramer G, Viall S, Summar, ML. Incidence of maple syrup urine disease, propionic acidemia, and methylmalonic aciduria from newborn screening data. Mol Genet Metab Rep. 2018;15:106-09. https://doi.org/10.1016/j.ymgmr.2018.03.011.

3. Almasi T, Guey LT, Lukacs C, Csetneki K, Voko Z, Zelei T. Systematic literature review and meta-analysis on the epidemiology of propionic acidemia. Orphanet J Rare Dis. 2019;14:40. https://doi.org/10.1186/s13023-018-0987-z.

4. Centers for Disease Control and Prevention (CDC). National Center for Health Statistics, Births and Natality. Births: Final Data for 2019. https://www.cdc.gov/nchs/fastats/births.htm. [Accessed June 16, 2021]

5. US Census Bureau. Quick Facts, Population, Census, April 1, 2020. https://www.census.gov/quickfacts/fact/table/US/PST045219. [Accessed June 16, 2021]

6. Surtees RA, Matthews EE, Leonard JV. Neurologic Outcome of Propionic Acidemia. Pediatr Neurol. 1992;8:333-7. https://doi.org/10.1016/0887-8994(92)90085-D

7. Shchelochkov OA, Carrillo N, Venditti C. Propionic Acidemia.2012 May 17 [updated 2016 Oct 6]. In: GeneReviews [Internet]. Seattle (WA): University of Washington, Seattle; 1993-2021. PMID: 22593918. https://www.ncbi.nlm.nih.gov/books/NBK92946/. [Accessed June 2, 2021]

8. Shchelochkov OA, Manoli I, Juneau P, Sloan JL, Ferry S, Myles J, Schoenfeld M, Pass A, McCoy S, Van Ryzin C, Wenger O, Levin M, Zein W, Huryn L, Snow J, Chlebowski C, Thurm A, Kopp JB, Chen KY, Venditti CP. Severity modeling of propionic acidemia using clinical and laboratory biomarkers. Genet Med. 2021;May 18. doi: 10.1038/s41436-021-01173-2. Online ahead of print. PMID: 34007002.

9. Chandler RJ, Chandrasekaran S, Carrillo-Carrasco N, Senac JS, Hofherr SE, Barry MA, Venditti CP. Adeno-associated virus serotype 8 gene transfer rescues a neonatal lethal murine model of propionic acidemia. Hum Gene Ther. 2011;22:477-81. doi: 10.1089/hum.2010.164.

10. Guenzel AJ, Hofherr SE, Hillestad M, Barry M, Weaver E, Venezia S, Kraus JP, Matern D, Barry MA. Generation of a hypomorphic model of propionic acidemia amenable to gene therapy testing. Mol Ther. 2013;21:1316-23. Doi: 10.1038/mt.2013.68.

11. Guenzel AJ, Hillestad ML, Matern D, Barry MA. Effects of adeno-associated virus serotype and tissue-specific expression on circulating biomarkers of propionic acidemia. Hum Gene Ther. 2014;25:837-43. Doi: 10.1089/hum.2014.012.

12. Guenzel AJ, Collard R, Kraus JP, Matern D, Barry MA. Long-term sex-biased correction of circulating propionic acidemia disease markers by adeno-associated virus vectors. Hum Gene Ther. 2015;26:153-60. Doi: 10.1089/hum.2014.126.

13. Feuchtbaum L, Carter J, Dowray S, Currier RJ, Lorey F. Birth prevalence of disorders detectable through newborn screening by race/ethnicity. Genet Med. 2012;14:937-45. doi: 10.1038/gim.2012.76.

14. Frazier DM, Millington DS, McCandless SE, Koeberl DD, Weavil SD, Chaing SH, Muenzer J. The tandem mass spectrometry newborn screening experience in North Carolina: 1997–2005. J Inherit Metab Dis. 2006;29(1):76–85. Doi:10.1007/s10545-006-0228-9.

15. Therrell BL Jr, Lloyd-Puryear MA, Camp KM, Mann MY. Inborn errors of metabolism identified via newborn screening: Ten-year incidence data and costs of nutritional

interventions for research agenda planning. Mol Genet Metab. 2014;113(1):14–26. Doi: 10.1016/j.ymgme.2014.07.009.

16. Weisfeld-Adams JD, Morrissey MA, Kirmse BM, Salveson BR, Wasserstein MP, McGuire PJ, Sunny S, Cohen-Pfeffer JL, Yu C, Caggana M, Diaz GA.. Newborn screening and early biochemical follow-up in combined methylmalonic aciduria and homocystinuria, cblC type, and utility of methionine as a secondary screening analyte. Mol Genet Metab 2010;99(2):116–123. doi: 10.1016/j.ymgme.2009.09.008.

17. Comeau AM, Larson C, Eaton RB. Integration of new genetic diseases into statewide newborn screening: New England experience. Am J Med Genet C Semin Med Genet. 2004;125C(1):35–41. doi: 10.1002/ajmg.c.30001.

18. Naylor EW, Chace DH. Automated tandem mass spectrometry for mass newborn screening for disorders in fatty acid, organic acid, and amino acid metabolism. J Child Neurol 1999;14 Suppl 1:S4–S8. PMID:10593560. https://doi.org/10.1177%2F0883073899014001021.

19. Chace DH, DiPerna JC, Kalas TA, Johnson RW, Naylor EW. Rapid diagnosis of methylmalonic and propionic acidemias: quantitative tandem mass spectrometric analysis of propionylcarnitine in filter-paper blood specimens obtained from newborns. Clin Chem. 2001;47(11):2040–4. PMID: 11673377.

20. Zytkovicz TH, Fitzgerald EF, Marsden D, Larson CA, Shih VE, Johnson DM, et al. Tandem mass spectrometric analysis for amino, organic, and fatty acid disorders in newborn dried blood spots: a two-year summary from the New England newborn screening program. Clin Chem. 2001;47(11):1945–55.

APPENDIX A

Nucleotide Sequence of AAV9-hPCCA: The complete sequence of AAV9-hPCCA drug product, including the codon optimized human PCCA gene and the different AAV9 cassette elements with the corresponding ITRs and promoter.

[The complete sequence of the drug product]

Office of Orphan Products Development
Food and Drug Administration
WO32-5295
10903 New Hampshire Avenue
Silver Spring, MD 20993

ORPHAN DRUG DESIGNATION ACKNOWLEDGEMENT LETTER

National Institutes of Health, National Center for Advancing Translational Sciences
9800 Medical Center Dr.
Rockville, Maryland 20850

Attention: *[Name and contact information for primary contact]*

Dear *[Primary Contact Name]*:

This letter acknowledges receipt of your orphan-drug designation request submitted pursuant to section 526 of the Federal, Food, Drug, and Cosmetic Act (21 U.S.C. 360bb) for the following:

Name: Adeno-Associated Virus 9 human Propionyl-CoA Carboxylase, alpha subunit (AAV9-hPCCA)

Disease or Condition: Treatment of propionic acidemia (PA) resulting from a deficiency of propionyl-CoA carboxylase, alpha subunit (PCCA)

Date of request: 7/2/2021

Date of receipt: 7/1/2021

Designation request number: DRU-2021-*[####]*

OOPD will correspond with you after we have completed our review of your designation request. This may take up to 90 days. All communications concerning the request should be identified with the above designation request number.

OOPD will respond to your request for designation by email. Please provide updated contact information if and when it changes. OOPD will assume that all emails from your representatives, or email addresses provided as a point of contact in your request, are FDA secure when responding to those email addresses. Transmissions to and from the FDA using FDA secure email addresses are encrypted. You can establish a secure email address link to FDA by sending a request to *[email address]*. There may be a

fee to a commercial enterprise for establishing a digital certificate as part of the set-up process before emails can be sent to FDA encrypted.

Should you have any questions, please contact our Office by phone at *[phone number]* or by email at *[email address]*.

Sincerely,

{See appended electronic signature page}

Office of Orphan Products Development

[Digital signature of FDA official]

Digitally signed by *[Name of FDA official]*

Date: 7/2/2021 8:18 AM EDT
GUID: *[#####]*

Office of Orphan Products Development
Food and Drug Administration
WO32- 5295
10903 New Hampshire Avenue
Silver Spring, MD 20993

National Institutes of Health
National Center for Advancing Translational Sciences
9800 Medical Center Dr.
Rockville, Maryland 20850

Attention: *[Name and contact information for primary contact]*

Re: Designation request # DRU-2021-*[####]*
Dated: 7/1/2021
Received: 7/1/2021

Dear *[Primary Contact Name]*:

This letter responds to your request for orphan-drug designation of adeno-associated virus 9 human propionyl-CoA carboxylase, alpha subunit (AAV9-hPCCA) for "treatment of propionic acidemia resulting from a deficiency of propionyl-CoA carboxylase, alpha subunit."

Pursuant to section 526 of the Federal Food, Drug, and Cosmetic Act (21 U.S.C. 360bb), your orphan-drug designation request of adeno-associated virus 9 human propionyl-CoA carboxylase, alpha subunit (AAV9-hPCCA) is granted for *treatment of propionic acidemia*. Please be advised that it is the active moiety or principal molecular structural features of the drug[1] and not the formulation of the drug that is designated. Please note that the designation granted is broader than the indication proposed in your designation request. Treatment of propionic acidemia resulting from a deficiency of propionyl-CoA carboxylase, alpha subunit is within the scope of this orphan-drug designation.

If your drug receives marketing approval for an indication broader than what is designated, it may not be entitled to exclusive marketing rights under section 527 (21 U.S.C.360cc). Therefore, prior to submission of your marketing application, we request that you compare the drug's orphan designation with the proposed marketing indication and submit additional information to amend the orphan-drug designation if warranted. 21 CFR 316.26.

[1] The term "drug" in this letter includes drug and biological products.

If the same drug is approved for the same indication before you obtain marketing approval of your drug, you will have to demonstrate that your drug is clinically superior to the already approved same drug in order to obtain orphan-drug exclusivity. Failure to demonstrate clinical superiority over the already approved same drug will result in your drug not receiving orphan-drug exclusivity. 21 CFR 316.34(c).

You must submit to the Office of Orphan Products Development a brief progress report of drug development within 14 months after this date and annually thereafter until marketing approval. 21 CFR 316.30.

Please notify this office within 30 days of submitting a marketing application for the drug's designated use. Once your marketing application is approved, please contact our office at 301-796-8660 to assess eligibility for orphan-drug exclusivity.

Should you have any questions, please contact our office by phone at 301-796-8660 or by email at orphan@fda.hhs.gov. Congratulations on obtaining your orphan-drug designation.

Sincerely,

{See appended electronic signature page}

[Name and Title of FDA Official]
Office of Orphan Products Development

[Signature of FDA official]

[digital signature information]
Date: 9/27/2021 10:36 AM EDT
[digital signature information]
