# Supplementary material for: Successfully Navigating Food and Drug Administration Orphan Drug and Rare Pediatric Disease Designations for AAV9-hPCCA Gene Therapy: The National Institutes of Health Platform Vector Gene Therapy Experience
Source: Hum Gene Ther. 2023 Mar 20;34(5-6):217–27. doi: 10.1089/hum.2022.232 (PMC10031144; doi:10.1089/hum.2022.232)
Supplement: Supplemental data [file Supp_FileS2.pdf]

About the AAV9-hPCCA Rare Pediatric Disease Designation Request Documents

The following documents are communications between the National Institutes of Health (NIH) National Center for Advancing Translational Sciences (NCATS) and the US Food and Drug Administration (FDA) Office of Orphan Product Development (OOPD) regarding a Rare Pediatric Disease (RPD) designation request to OOPD. The disease for this RPD designation is propionic acidemia (PA) resulting from a deficiency of Propionyl-CoA Carboxylase, alpha subunit (PCCA), and the RPD product is AAV9-hPCCA (NCATS-BL0746), a gene therapy under development as part of the Platform Vector for Gene Therapy (PaVe-GT) program.

PaVe-GT is a pilot project that will test whether the efficiency of gene therapy trial startup can be significantly improved by using a standardized process across gene therapies for four different rare diseases. An important goal of PaVe-GT is to share project results and lessons learned with the public in such a way that the information is useful to any party interested in developing a gene therapy efficiently. Specifically, we will make information and results from the PaVe-GT program publicly available in as timely a manner as possible. This includes toxicology and biodistribution data, Investigational New Drug filings and communications with the U.S. Food and Drug Administration, and other study documents. To ensure access to the latest learnings, please visit the PaVe-GT website, subscribe to project updates, and explore the full set of available resources at pave-gt.ncats.nih.gov.

Some portions of this document—primarily sections that are highly specific to *PCCA*-PA and therefore not relevant to other AAV gene therapy efforts—have been edited, redacted or abridged to improve the clarity of materials, and/or support other project objectives. Modified sections are typically identified with italics, brackets, and highlight, *[as shown here]*. The text within the brackets describes the original content.

Disclaimer: NCATS and NIH provides no warranties, representations or guarantees that PaVe-GT Resources will work for any given project or disease condition. Further, NIH disclaims any liability and provides no indemnification. For a full list of terms and conditions for use of PaVe-GT resources, visit pave-gt.ncats.nih.gov.

Table of Contents

RPD Designation Request Cover Letter **2**

RPD Designation Request **3**

FDA Acknowledgment Letter **29**

FDA Response Letter **31**

June 17, 2022

Attention: Office of Orphan Products Development, Rare Pediatric Disease Designation Program

Re: Rare Pediatric Disease Designation request for Adeno-Associated Virus 9 human Propionyl-Coenzyme A (CoA) Carboxylase, alpha subunit (AAV9-hPCCA) for the treatment of patients with propionic acidemia (PA) resulting from a deficiency of Propionyl-CoA Carboxylase, alpha subunit (PCCA).

Dear *[Name of Point of Contact within OOPD]*:

Pursuant to Section 908 of the Food and Drug Administration Safety and Innovation Act (FDASIA), the National Institutes of Health (NIH), National Center for Advancing Translational Sciences (NCATS), requests designation of Adeno-Associated Virus 9 human Propionyl-CoA Carboxylase, alpha subunit (AAV9-hPCCA) as a Rare Pediatric Disease (RPD) drug product for the treatment of patients with propionic acidemia (PA) resulting from a deficiency of Propionyl-CoA Carboxylase, alpha subunit (PCCA). AAV9-hPCCA has been granted an Orphan Drug Designation (ODD) by FDA for the treatment of *PCCA*-related PA on September 27, 2021 (Designation request # *DRU-2021-[####]*). In addition, we have included a pdf attachment, titled "NCATS AAV9-hPCCA Full References," consisting of full articles for all referenced research citations in our application.

I, *[primary contact name]*, will serve as the primary contact on this submission, and *[name and title of the secondary contact]* will serve as secondary contact (*[contact information for the secondary contact]).*

For any questions regarding this submission, please contact me at *[contact information for the primary contact]*, including *[the alternate contact]* listed above.

Sincerely,

[The name, title and contact information for the primary contact]

National Institutes of Health (NIH), National Center for Advancing Translational Sciences (NCATS)

Adeno-Associated Virus 9 human Propionyl-CoA Carboxylase, alpha subunit (AAV9-hPCCA) for the Treatment of Patients with Propionic Acidemia Resulting from a Deficiency of Propionyl-CoA Carboxylase, Alpha Subunit (PCCA)

Rare Pediatric Disease Designation Request

June 17, 2022

- CONFIDENTIAL -

This document and its contents are the property of and confidential to NCATS. Any unauthorized copying or use of this document is prohibited.

Table of Contents

[The page numbers in the table of contents below have been adjusted from their original values in the request to be consistent with their page numbers in the PaVe-GT AAV9-hPCCA Rare Pediatric Designation Materials pdf.]

Section **Page**

List of Abbreviations **6**

1 Rare Pediatric Disease Designation Request Statement **7**

2 Administrative Information **8**

2.1 Sponsor 8

2.2 Primary and Alternate Contacts 8

2.3 Drug Name 8

2.3.1 Chemical Name - Drug Substance 8

2.3.2 Generic/Trade Name - Drug Product 8

2.4 Manufacturer's Name and Address 9

2.4.1 Drug Substance Manufacturer 9

2.4.2 Drug Product Manufacturer 9

3 Description of Rare Disease or Condition, Proposed Indication, and Need for Therapy **9**

3.1 Description of Rare Disease or Condition 9

3.2 Proposed Indication and Use of AAV9-hPCCA 11

3.3 Reasons Why Such Therapy Is Needed 12

4 Description of AAV9-hPCCA and Scientific Rationale for Use **13**

4.1 Description of AAV9-hPCCA 13

4.1.1 Drug Product Naming Convention 13

4.1.2 Drug Product Vector Design 13

4.1.3 Drug Product Description, Mechanism of Action and Route of Administration 13

4.2 Scientific Rationale for the Use of AAV9-hPCCA in the Rare Disease or Condition 14

4.2.1 Clinical Efficacy of AAV9-hPCCA 14

4.2.2 Nonclinical Efficacy of AAV9-hPCCA 15

5 Orphan Drug Status **19**

6 Patient Subset Considerations and Medical Plausibility of the Chosen Subset 20

7 Regulatory Status and Marketing History 20

8 Documentation of Patient Population Size 20

9 Rare Pediatric Disease Designation Justification 24

10 References 25

LIST OF ABBREVIATIONS

AAV	Adeno-Associated Virus
AAV9	Adeno-Associated Virus serotype 9
AAV9-hPCCA	Adeno-Associated Virus serotype 9 human Propionyl-CoA Carboxylase, alpha subunit
Cas9	CRISPR associated protein 9
CoA	Coenzyme A
CRISPR	Clustered regularly interspaced short palindromic repeats
CRM	Cross Reactive Material
DBS	Dried Blood Spots
FDASIA	Food and Drug Administration Safety and Innovation Act
IER	Inborn Errors of Metabolism
ITR	Inverted Terminal Repeat
LT	Liver Transplantation
2-MC	Total 2-Methylcitrate
MCEE	D-methylmalonyl-CoA epimerase
MENA	Middle-East and North Africa
MMUT	Methylmalonyl-CoA mutase
mRNA	Messenger Ribonucleic Acid
NBS	Newborn Screening
NCATS	National Center for Advancing Translational Sciences
NHGRI	National Human Genome Research Institute
NIH	National Institutes of Health
NHS	Natural History Study
OAA	Oxaloacetic acid
P	Postnatal Day
PA	Propionic Acidemia
PBS	Phosphate Buffered Saline
PCC	Propionyl-CoA Carboxylase (EC 6.4.1.3)
Pcca	Propionyl-CoA Carboxylase, alpha subunit gene (mouse)
PCCA	Propionyl-CoA Carboxylase, alpha subunit protein
PCCA	Propionyl-CoA Carboxylase, alpha subunit gene (human)
PCCB	Propionyl-CoA Carboxylase, beta subunit gene (human)
POC	Proof of Concept
qPCR	Quantitative Polymerase Chain Reaction
RNA	Ribonucleic acid
ROA	Route of Administration
RPD	Rare Pediatric Disease
US	United States
vg	vector genome
WES	Whole-exome Sequencing
WT	Wild Type

1 RARE PEDIATRIC DISEASE DESIGNATION REQUEST STATEMENT

Pursuant to Section 908 of the Food and Drug Administration Safety and Innovation Act (FDASIA),[1] the National Institutes of Health (NIH), National Center for Advancing Translational Sciences (NCATS) requests designation of Adeno-Associated Virus serotype 9 human Propionyl-CoA Carboxylase, alpha subunit (AAV9-hPCCA) as a Rare Pediatric Disease (RPD) product for treatment of patients with propionic acidemia (PA) resulting from a deficiency of Propionyl-CoA Carboxylase, alpha subunit (PCCA). AAV9-hPCCA has been granted an Orphan Drug Designation (ODD) by FDA for the treatment of *PCCA*-related PA on September 27, 2021 (Designation request # DRU-2021-*[####]*).

PA is serious and life-threatening disorder caused by deleterious mutations in either the *PCCA* or *PCCB* genes, which encode for the α and β subunits, respectively, of the multimeric enzyme propionyl-CoA carboxylase (PCC). PCC is a ubiquitously expressed enzyme that plays a role in the normal breakdown of branched chain amino acids (isoleucine and valine), methionine, threonine, gut-derived propionate, and odd chain fatty acids. PCC deficiency leads to episodes of metabolic decompensation/acidemia resulting in acute neurological crises, cardiomyopathy, and other serious clinical manifestations. There are no FDA approved therapies for the treatment of PA.

PA is a rare disease in the United States (US). NIH NCATS received an ODD for AAV9-hPCCA for the treatment of *PCCA*-related PA based on review and analysis of published studies in the medical literature relevant to the US population, primarily based on birth incidence calculated from State newborn screening (NBS) data, as well as an NIH-conducted natural history study (NHS) and expert opinion.[2,3,4] This analysis led to an estimated population size of 50 to 3,962 *PCCA*-related PA patients living in the US in 2021 and qualifying *PCCA*-related PA as a rare disease.

PCCA-related PA is a genetic disorder that most commonly presents as neonatal PA within the first few days of life with hyperammonemia, poor feeding, vomiting, irritability, and lethargy.[5] Without treatment, these infants may develop neonatal encephalopathy, seizures, coma, and respiratory failure, which if left untreated can result in death. The less common late-onset form typically presents in early childhood (<1 year of age) with acute decompensation precipitated by metabolic stressors, such as infection, injury or surgery.[6] Infrequently, PA may present in late childhood with dilated cardiomyopathy or acute psychosis without prior known history of metabolic decompensations.[5] Universal NBS in the US can identify most affected patients. However, even with available treatments, mainly consisting of dietary restrictions and L-carnitine supplementation, the long-term prognosis for survival in severely affected patients is poor. Most patients will suffer from recurrent metabolic instability precipitated by periods of metabolic stress, and can develop chronic progressive multisystemic complications, including cardiomyopathy. Over the past several decades, it has been recurrently noted that PA patients

with an early and severe clinical course experience increased mortality and disease associated morbidity throughout childhood and adolescence.[7]

Thus, based on available evidence, *PCCA*-related PA meets the criteria for designation as a RPD including: 1) *PCCA*-related PA is a serious or life-threatening disease in which the serious or life-threatening manifestations primarily affect individuals aged from birth to 18 years; and 2) *PCCA*-related PA is a rare disease. We therefore request that AAV9-hPCCA be designated as a Rare Pediatric Disease Product for the treatment of *PCCA*-related PA.

2 ADMINISTRATIVE INFORMATION

2.1 Sponsor

National Institutes of Health (NIH), National Center for Advancing Translational Sciences (NCATS)

9800 Medical Center Dr.
Rockville, MD 20850

2.2 Primary and Alternate Contacts

Primary Contact
[The primary contact and their contact information]

Alternate Contact
[The alternate contact and their contact information]

2.3 Drug Name

2.3.1 Chemical Name - Drug Substance

Adeno-Associated Virus 9 human Propionyl-CoA Carboxylase, alpha subunit (AAV9-hPCCA)

2.3.2 Generic/Trade Name - Drug Product

AAV9-hPCCA is an Adeno-Associated Virus 9 (AAV9) vector expressing a functional human codon optimized cDNA encoding the Propionyl-CoA Carboxylase, alpha subunit (*PCCA*), under control of *[a specific promoter.]* Details describing the drug product are found in ***Section 4.1.***

Additional Names/Identifiers: NCATS-BL0746

[Diagram of the components of the AAV9-hPCCA vector]

2.4 Manufacturer’s Name and Address

2.4.1 Drug Substance Manufacturer

[The name, address, and phone number of the drug substance manufacturer]

2.4.2 Drug Product Manufacturer

[The name, address, and phone number of the drug product manufacturer]

3 DESCRIPTION OF RARE DISEASE OR CONDITION, PROPOSED INDICATION, AND NEED FOR THERAPY

3.1 Description of Rare Disease or Condition

Clinical Manifestations

Propionic acidemia (PA) is a rare inborn error of metabolism resulting from deleterious variants in the *PCCA* or *PCCB* genes leading to impaired activity of propionyl-CoA carboxylase (PCC). PCC is a ubiquitously expressed mitochondrial enzyme whose function is closely linked to energy production. It plays a role in the metabolism of the branched chain amino acids valine, methionine, isoleucine and threonine, gut-derived propionate, and odd-chain fatty acids. The clinical course of PA is characterized by chronic multiorgan dysfunction punctuated by episodes of metabolic instability due to metabolic acidosis, ketonuria, hyperammonemia, and hypoglycemia leading to emergency room visits and hospitalizations as seen in ***Figure 1***. Most frequently, PA presents in the neonatal period with hyperammonemia, poor feeding, vomiting, irritability, and lethargy. Without treatment, these infants may develop neonatal encephalopathy, seizures, coma, and respiratory failure, which if left untreated can result in death. Universal NBS implemented in the US can identify most affected infants. Infrequently, NBS fails to identify affected newborns, usually infants homozygous for the “mild” Amish-Mennonite *PCCB* allele (*PCCB*:c.1606A>G, p.Asn536Asp). Such milder cases can present later in life with life-threatening dilated cardiomyopathy.

Figure 1. Clinical and Laboratory Manifestations of PA

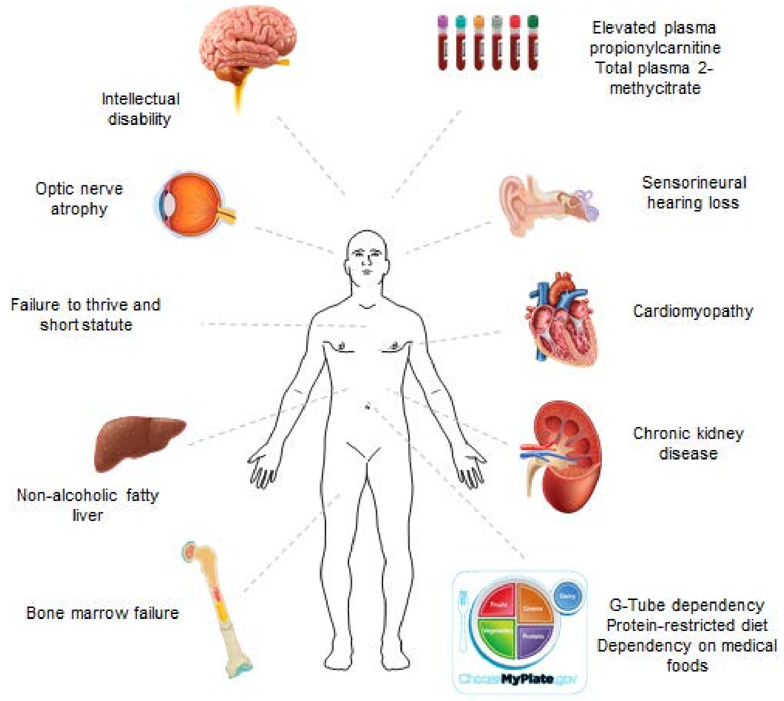

Image source: Shchelochkov, O.A., Manoli, I., Juneau, P. *et al.* Severity modeling of propionic acidemia using clinical and laboratory biomarkers. *Genet Med* **23,** 1534–1542 (2021). https://doi.org/10.1038/s41436-021-01173-2

PA is a rare disorder with birth prevalence varying widely by region. Worldwide, the birth incidence has been reported as high as 1:1000 and as low as 1:500,000. The estimated birth incidence of PA in the US ranges from 0.13 to 1.2 affected infants per 100,000 births.[2,3,4] (Please see ***Section 8*** for detailed discussion on birth incidence).

The long-term prognosis for survival in severely affected patients is poor, which was first illustrated in the 1990s by an early and relatively large (for the disease prevalence) single center study of 20 patients with PA treated at a tertiary care center.[7] This study showed that patients who presented in the first week of life (11 patients) largely perished by the age of 6 years. Even with treatment, patients who survived suffered from recurrent metabolic instability often precipitated by periods of metabolic stress, such as infections, and can develop chronic progressive multisystemic complications, including cardiomyopathy. Over the subsequent decades, it has been recurrently noted that PA patients with an early and severe clinical course experience increased mortality and disease associated morbidity.[5] The recalcitrant nature of the disorder to conventional medical management, including the dietary restriction of amino acid precursors, L-carnitine supplementation, and administration of oral metronidazole to reduce the generation of propionic acid by intestinal bacteria, has led to the implementation of elective liver transplantation (LT) as an experimental surgical treatment for PA. While not curative of all aspects of disorder, successful LT in the setting of PA may improve metabolic stability and

protection from early death, and therefore represents a clinical benchmark for gene replacement or other approaches that might increase hepatic PCC expression and activity.

Pathophysiology

PCC is a ubiquitously expressed, heteropolymeric mitochondrial enzyme involved primarily in the catabolism of propiogenic amino acids, particularly isoleucine, valine, methionine, and threonine, as well as odd-chain fatty acids. The enzyme is composed of α- and β-subunits encoded by their respective genes, *PCCA* and *PCCB*, and PA is caused by biallelic pathogenic variants in either gene. PCC catalyzes the first step in the conversion of propionyl-CoA to D-methylmalonyl-CoA in the pathway of propionate oxidation, depicted in ***Figure 2***. The formation of 2-methylcitrate (2-MC), an important biomarker likely generated through the condensation of oxaloacetic acid (OAA) and propionyl-CoA is also noted, as are downstream enzymatic steps in the pathway, including D-methylmalonyl-CoA epimerase (MCEE) and methylmalonyl-CoA mutase (MMUT), in the metabolism of propionyl-CoA into the citric acid (Krebs) cycle.

Figure 2. Catabolism of Propionyl-CoA.

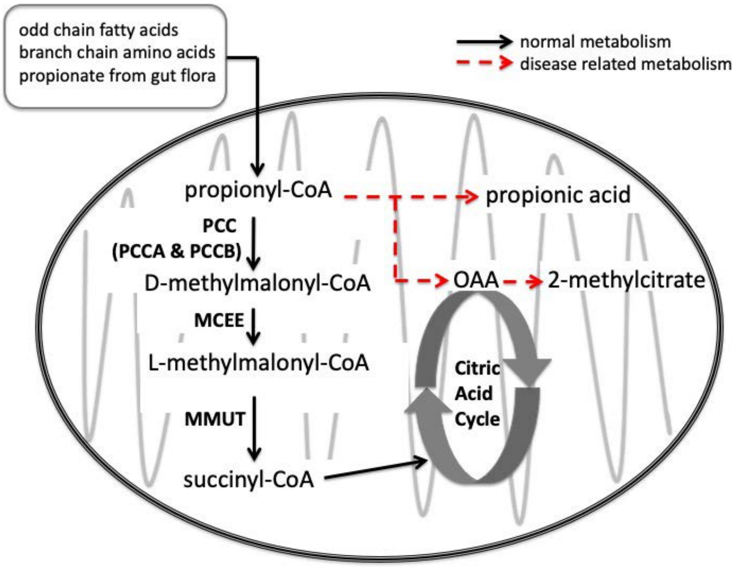

3.2 Proposed Indication and Use of AAV9-hPCCA

The proposed clinical use of AAV9-hPCCA is for the treatment of patients with PA resulting from a deficiency of PCC due to deleterious variants in PCCA. Briefly, AAV9-hPCCA is an AAV9 vector expressing a functional human codon optimized cDNA encoding PCCA, under control of *[a specific promoter.]* In a newly generated knockout murine model designated *Pcca*$^{-/-}$, AAV9-hPCCA demonstrated efficacy. The *Pcca*$^{-/-}$ animals have no detectable Pcca protein and display immediate neonatal lethality, akin to the phenotype of the human disorder.

Upon retro-orbital injection of AAV9-hPCCA in these animals, *[description of dose responses]* was observed. In an initial biodistribution study of AAV9-hPCCA treated *Pcca*$^{-/-}$, *PCCA* mRNA and PCCA protein were found in both target tissues, heart and liver, as analyzed by qPCR and Western blotting, respectively. The *Pcca*$^{-/-}$ animals also display higher levels of the 2-MC biomarker in the plasma which was substantially reduced upon administration of AAV9-hPCCA.

3.3 Reasons Why Such Therapy Is Needed

There are currently no FDA-approved therapies for PA and novel therapies are urgently needed to prevent irreversible disease complications and improve patient management. PA manifests life-threatening debilitating illness in early childhood and is poorly responsive to available medical therapy. Adult PA patients have high prevalence of severe kidney and heart disease, and irreversible disease complications to sensory organs.[5] Current standards of care include strict adherence to low-protein diet with and without poorly palatable medical foods, and supplementation with levocarnitine. Other treatments may apply if patients develop severe complications of PA, such as, the use of cardiac medications in patients with cardiomyopathy or antiepileptic drugs in PA patients with epilepsy.[7] The long-term prognosis for survival in severely affected patients is poor as illustrated by an early and relatively large (for the disease prevalence) single center study of 20 patients with PA treated at a tertiary care center: Those who presented in the first week of life (11 patients) died by the age of 6 years.[7] Over the decades, it has been recurrently noted that PA patients with an early and severe clinical course experience increased mortality and disease associated morbidity.[5]

Some PA patients, who experience frequent hospitalizations due to metabolic instability and/or who develop dilated cardiomyopathy may undergo liver transplantation (LT) and require life-long immunosuppression. While not curative of all aspects of the disorder, successful LT in the setting of PA provides restoration of metabolic stability and protection from early death, and therefore represents a clinical benchmark for gene replacement or addition approaches that might increase hepatic PCC expression and activity. Although the post-LT status in PA remains to be fully elucidated, some patients have developed renal disease and even cardiomyopathy after successful LT. An additional and potentially fatal complication, presumed secondary to chronic exposure to immune suppression, has been the development of post-transplant lymphoproliferative disease. Therefore, while LT can decrease frequency of metabolic decompensations, it clearly carries immediate/acute (post-surgical) and chronic/subacute risks. In theory, these risks could be mitigated or perhaps eliminated by successful liver-directed gene therapy with AAV9-hPCCA, which we propose as a single IV infusion aiming to restore therapeutic levels of PCCA in the liver and heart.

4 DESCRIPTION OF AAV9-HPCCA AND SCIENTIFIC RATIONALE FOR USE

4.1 Description of AAV9-hPCCA

4.1.1 Drug Product Naming Convention

Adeno-Associated Virus 9 human Propionyl-CoA Carboxylase, alpha subunit (AAV9-hPCCA)

4.1.2 Drug Product Vector Design

AAV9-hPCCA is an AAV9 vector expressing a functional human codon optimized cDNA encoding *PCCA*, under control of *[a specific promoter]*. In humans, endogenous PCCA protein is ubiquitously expressed, therefore we designed a therapeutic transgene cassette with a constitutive promoter to enable wide expression and selected the AAV9 capsid to further enable hepatic and cardiac transduction. A schematic of the vector transgene, description of cassette features summarizing the salient features of the AAV vector and the vector map is presented in ***Figure 3*** and ***Figure 4***. The complete nucleotide sequence of AAV9-hPCCA is included as ***Appendix A.***

Figure 3. A Schematic of the Vector Transgene.

[Diagram of the components of the AAV9-hPCCA vector, previously included under 2.4.2]

[Table describing basic elements of the cassette including: the length of the inverted terminal repeats, whether there is an enhancer, what is the promoter, if there is an intron, if there is a 5' untranslated sequence, the nature of the therapeutic gene (e.g. natural or codon-adjusted), if there is a 3' untranslated sequence included, the polyadenylation signal, 3' inverted terminal repeat, comments about genome configuration (e.g. single strand or double strand) and additional remarks regarding the regulatory sequences and/or transgene, such as microRNA binding sites.]

Figure 4. Vector Map.

[A map of the plasmid used to produce the therapeutic transgene. In addition to noting major components of the therapeutic transgene, other features such as antibiotic resistance, origin of replication, and salient details surrounding the plasmid backbone were included. The size of the transgene and constitutive elements were denoted.]

4.1.3 Drug Product Description, Mechanism of Action and Route of Administration

AAV9-hPCCA is a biologic modality for introducing corrected DNA into PA patients with

biallelic pathogenic variants in the *PCCA* gene. The gene is delivered using an AAV virus, serotype AAV9, a modified virus that is not infectious in humans but maintains its natural ability to deliver genetic material into cells. The AAV9-hPCCA will introduce a normal copy of the human *PCCA* gene and help restore the normal function of the PCC enzyme in the catabolism of propionyl-CoA, as described in ***Figure 2***, ***Section 3.1***. AAV9-hPCCA will be stored at -80°C and delivered in patients as an intravenous (IV) solution using the peripheral IV route of administration (ROA). The final formulation for the drug product has not been determined but the formulation for an initial batch for preclinical *in vivo* animal studies was *[formulation details]*.

4.2 Scientific Rationale for the Use of AAV9-hPCCA in the Rare Disease or Condition

PA is caused by biallelic pathogenic variants in either the *PCCA* or *PCCB* gene. It is caused by a deficiency of PCC, a ubiquitously expressed, heteropolymeric mitochondrial enzyme involved primarily in the catabolism of propionogenic amino acids. The enzyme is composed of α- and β-subunits encoded by their respective genes, *PCCA* and *PCCB*. PCC catalyzes the first step in the conversion of propionyl-CoA to D-methylmalonyl-CoA in the pathway of propionyl-CoA oxidation, depicted in ***Figure 2***, ***Section 3.1***. Replacement of the PCCA enzyme through delivery of AAV9-hPCCA gene therapy is expected to help normalize the metabolic defects associated with PA.

4.2.1 Clinical Efficacy of AAV9-hPCCA

To date, no clinical data have been collected with AAV9-hPCCA for the treatment of patients with PA resulting from a deficiency of PCCA protein. However, as stated previously, PA patients that have undergone successful LT demonstrate improved metabolic stability and protection from early death, and therefore represents a clinical benchmark for gene replacement therapy that may lead to increased hepatic PCC expression and activity.

There is a lack of natural history longitudinally evaluating PA biomarkers and their association with outcomes corresponding to survival, functioning and disease burden. To close this gap, the Organic Acidemia Section of National Human Genome Research Institute (NHGRI) initiated a NHS of the PA population (National Clinical Trial # NCT02890342).[8] The study is being conducted at the NIH Clinical Center and has enrolled 44 participants ages 2 years and older (2.5 years – 54 years of age). The cohort features a genetically heterogeneous PA population, a high proportion of adult patients (~40%), and a subset of liver- and kidney-transplanted PA participants. A recent publication has identified candidate biomarkers and surrogate endpoints in this cohort.[8]

4.2.2 Nonclinical Efficacy of AAV9-hPCCA

***In Vitro* Studies in Human Liver (*[cell line name]*) PCCA Knockout (KO) Cell Line**
Infection of an engineered human liver *[cell line name]*/PCCA KO cell line incubated with varying concentrations (*[list of concentration values]*) of AAV9-hPCCA showed *[pattern of expression]* of PCC enzyme by Western Blotting upon probing with a PCC specific antibody that was not seen in the control (uninfected KO cells) ***(Figure 5).*** In addition, cell lysates from *[cell line name]*/PCCA KO infected cells also showed *[description of dose response]* in PCC enzyme activity as measured by a *[method]* that quantitates the conversion of propionyl-CoA to methylmalonyl-CoA (not presented). The *[pattern of]* PCC protein expression observed after AAV9-hPCCA infection is consistent with the *in vivo* *[pattern of]* expression of PCC protein measured in liver extracts from $Pcca^{-/-}$ mice after retroorbital injection of AAV9-hPCCA (***Figure 8***).

Figure 5. AAV9-hPCCA *In Vitro* Infection Studies in Human Liver (*[cell line name]*) PCCA Knockout (KO) Cell Line

[Image: Western blot results]

A deletion mutation in *PCCA* was engineered in a human hepatocyte derived cell line (*[cell line name]*) to create the PCCA KO cell line and used to test the in vitro potency of AAV9-hPCCA applied at varying moiety of infections ranging from *[lower concentration value]* to *[higher concentration value]*. After 48 hours, the infected PCCA KO cells were harvested and lysed, and *[a quantity]* of total cellular protein was subjected to Western analysis using a PCCA polyclonal antibody. Beta-actin was used as a loading control. *[Description of dose response.]*

***In Vivo* Studies in a Lethal Mouse Model of PA Used to Investigate Effect of AAV9-hPCCA**
Gene therapy has been administered to different animal models of PA caused by PCCA deficiency and reduction in metabolite levels indicated therapeutic potential of gene therapy.[9, 10, 11, 12] However, the previous models of PA were made by creating large genetic deletions in the *Pcca* gene or relied on transgenic constructions and did not fully recapitulate the genetic mutations or disease phenotype commonly observed in humans. Hence, for testing of AAV gene therapy vectors for human translation, we generated mice using CRISPR/Cas9 genome editing to engineer mutations that are compatible with those seen in the patients, such as frameshift-stop and missense changes. The mutation, Pcca *[mutation name]*, caused by a deletion in the *Pcca* gene, is severe and null at the level of PCCA protein expression (cross reactive material, CRM-). Mice homozygous for this mutation ($Pcca^{-/-}$) were generated and recapitulate the neonatal lethal form of PA in humans. These mice perish in the immediate neonatal period (within *[period of time]*) without gene therapy treatment, and hence, are particularly useful to assay the efficacy of AAV9-hPCCA gene therapy for the treatment of PA. Data from the *in vivo* studies demonstrate that administration of AAV9-hPCCA vector rescues $Pcca^{-/-}$ neonatal mice by increasing animal survival and reducing levels of the pathological metabolite.

We have conducted a series of studies in a newly generated knockout mouse model, designated *Pcca*$^{-/-}$ and detailed below. The mice have no detectable PCCA protein and display immediate neonatal lethality, akin to the phenotype of the human disorder. A summary of the POC studies conducted in *Pcca* $^{-/-}$ neonatal mice after administration of AAV9-hPCCA thus far is presented below in ***Table 1***. The gene therapy shows a pronounced effect on the animal survival and reduction in the plasma levels of the pathological metabolite, 2-MC.

Table 1. Survival and Metabolic Phenotype Summary of *Pcca*$^{-/-}$ Animals Treated with Different Doses of AAV9-hPCCA

Treatment Group	Dose (vg/kg)	No. of *Pcca*$^{-/-}$ Mice	Mean Survival (Days) ± Standard Deviation	Metabolic Phenotype (2-MC)
Untreated	N/A	*[value]*	*[value]*	*[value]*
[Vehicle]	N/A	*[value]*	*[value]*	*[value]*
*AAV9-hPCCA	*[lower dose]*	*[value]*	*[value]*	*[value]*
*AAV9-hPCCA	*[mid dose]*	*[value]*	*[value]*	*[value]*
*AAV9-hPCCA	*[higher dose]*	*[value]*	*[value]*	*[value]*

<u>*Note:</u> Different lots of research grade AAV9-hPCCA were used for the *Pcca* $^{-/-}$ studies listed in the table above, but all contained the same therapeutic transgene, and all animals were dosed by retro-orbital injection on post-natal day P0-1.

Detailed Experimental Design and Results

For definitive testing of AAV gene therapy vectors for human translation, we created mice using CRISPR/Cas9 genome editing to engineer mutations that are compatible with those seen in the patients, such as frameshift-stop and missense changes. One mutation, Pcca$^{[mutation\ name]}$ in Exon *[value]* of the *Pcca* gene is severe and null at the level of protein expression (CRM-). Please see ***Table 2***.

Table 2. *Pcca*$^{-/-}$ Neonatal Knockout Mouse Model

Pcca exon Targeted	Pcca Protein Mutation	Homozygous phenotype
[value]	*[mutation name]*	Neonatal lethal CRM negative<br>Increased 2-MC

Building on many years of previous work with AAV gene therapy in related mouse models of methylmalonic acidemia, where early lethality is a uniform characteristic of the homozygous mutant phenotype, we initiated our studies to determine the therapeutic efficacy of AAV9-hPCCA delivered by retro-orbital plexus injection in newborn *Pcca*$^{-/-}$ mice. Because the untreated *Pcca*$^{-/-}$ mice experience *[percentage value]* lethality by P2 (***Figure 6***), we injected

AAV9-hPCCA via retro-orbital plexus to the systemic circulation, to recapitulate IV delivery, the anticipated ROA in humans.

Survival of AAV9-hPCCA Treated *Pcca*$^{-/-}$ Mice

Homozygous *Pcca*$^{-/-}$ males rescued by AAV9 gene delivery were mated with heterozygous females. All the pups obtained in the litter were treated with AAV9-hPCCA within a few hours after birth (at P0-1). For the survival study in the *Pcca*$^{-/-}$ mice, we tested three doses of AAV9-hPCCA, *[lower dose]* vg/kg (n=*[value]*), *[mid dose]* vg/kg (n=*[value]*) and *[higher dose]* vg/kg (n=*[value]*), administered via retro-orbital injections. To minimize stress on the newborns and mothers, the pups were not weighed before AAV9-hPCCA administration, the vector was dosed as vg/pup and approximated for vg/kg using the average body weight of *[weight in grams]* (n=*[value]*). The genotypes of all pups were not determined until after injection.

Table 3 below summarizes the doses administered to the homozygous *Pcca*$^{-/-}$ neonatal animals. Subsequently, survival was monitored, and the transgene expression was measured. At selected timepoints, biomarker responses (2-MC) and vector genome biodistribution were measured.

Table 3. List of AAV9-hPCCA Treated Neonatal *Pcca*$^{-/-}$ Mice.

Genotype	Treatment	Dose (vg/pup)	Dose (vg/kg)	Sex	Number of Mice
Pcca$^{-/-}$	None	N/A	N/A	Not Available	*[value]*
Pcca$^{-/-}$	*[Vehicle]*	N/A	N/A	Not Available	*[value]*
Pcca$^{-/-}$	AAV9-hPCCA	*[value]*	*[lower dose]*	M	*[value]*
Pcca$^{-/-}$	AAV9-hPCCA	*[value]*	*[lower dose]*	F	*[value]*
Pcca$^{-/-}$	AAV9-hPCCA	*[value]*	*[lower dose]*	Not Available	*[value]*
Pcca$^{-/-}$	AAV9-hPCCA	*[value]*	*[mid dose]*	M	*[value]*
Pcca$^{-/-}$	AAV9-hPCCA	*[value]*	*[mid dose]*	F	*[value]*
Pcca$^{-/-}$	AAV9-hPCCA	*[value]*	*[mid dose]*	Not Available	*[value]*
Pcca$^{-/-}$	AAV9-hPCCA	*[value]*	*[higher dose]*	M	*[value]*
Pcca$^{-/-}$	AAV9-hPCCA	*[value]*	*[higher dose]*	F	*[value]*

The results for the various dose cohorts of AAV9-hPCCA treated *Pcca*$^{-/-}$ mice are presented in ***Figure 6***. *[Description of dose(s) that provide rescue from neonatal lethality, and prolong the life of the mutant mice.]* The control *Pcca*$^{-/-}$ animals (untreated/*[vehicle]*) died shortly after birth. The animals dosed at *[lower dose]* had a mean survival of *[value]* days, those in the *[mid dose]*

vg/ kg cohorts survived for *[value]* days, and those in the highest dose group of *[higher dose]* had a mean survival of *[value]*.

Figure 6. Survival Curve of AAV9-hPCCA Treated *Pcca*$^{-/-}$ Mice.

[Graph of survival curve. The y axis of the graph is percent survival of PCCA-/- mice, from 0 to 100. The x axis is number of days. Each line represents a different experimental group/dose.]

Mice were treated with indicated doses of AAV9-hPCCA at P0-1. *[Vehicle]* and no treatment (untreated) were used as a control. The graph depicts the percent survival of different cohorts of animals ** *P*<0.0001, ** *P*<0.01 compared to survival of untreated vs treated *Pcca*$^{-/-}$ mice. *P* values were calculated using a Log_rank (Mantel-Cox) test.**

Measurement of Plasma 2-MC in AAV9-hPCCA Treated *Pcca*$^{-/-}$ Mice

Animals dosed with AAV9-hPCCA were bled to measure a biomarker response (at *[multiple timepoints]*), and then sacrificed to assess transgene expression and vector biodistribution. On *[early timepoint]*, the weights of the AAV9-hPCCA treated *Pcca*$^{-/-}$ mice were not different than treated control littermates (not presented). As illustrated in ***Figure 7***, treatment of *Pcca*$^{-/-}$ mice with AAV9-hPCCA resulted in a substantial reduction in the plasma levels of 2-MC as compared to the untreated animals at doses of *[list of applicable doses]*.

Figure 7. Plasma 2-MC Levels in AAV9-hPCCA Treated *Pcca*$^{-/-}$ Mice.

[Graph of Plasma 2-MC levels for untreated mice and treated mice at the lower, mid and higher dose]

Mean $\pm$ S.D. of the plasma 2-MC levels in *Pcca*$^{-/-}$ mice treated at birth with AAV9-hPCCA. **P*<0.01 for *Pcca*$^{-/-}$ treated vs untreated at the time of birth calculated by a two-sided, two tailed t-test.

Transgene Expression in AAV9-hPCCA Treated *Pcca*$^{-/-}$ Mice

After treatment with AAV9-hPCCA, the transgene and protein levels were assessed in the liver of a small number of *Pcca*$^{-/-}$ mice treated with *[two of the dose levels]* of AAV9-hPCCA. The *PCCA* mRNA was quantified by qPCR and the PCCA protein levels were measured by Western blotting at *[two timepoints]*. The mRNA levels were normalized to endogenous glyceraldehyde 3-phosphate dehydrogenase (*Gapdh*) expression and protein levels were normalized to endogenous beta actin levels. The AAV encoded *PCCA* mRNA varied between *[range of percentage values]* of WT *Pcca* levels and the protein expression was at ~*[value]*% of the wild-type murine PCCA level (***Figure 8***).

Figure 8. Hepatic *PCCA* mRNA and PCCA Protein Expression in AAV9-hPCCA Treated *Pcca*$^{-/-}$ Mice

[A clustered bar graph reporting percent of WT PCCA expression and relative PCCA mRNA expression across several experimental groups: WT; untreated Pcca $^{-/-}$; and treated Pcca $^{-/-}$ mice at specific doses and timepoints]

Quantitation (Mean ± S.D.) of *PCCA* mRNA and PCCA protein represented as a percentage of endogenous WT expression levels and normalized to beta-actin for protein levels and to *Gapdh* for mRNA expression.

Biodistribution in AAV9-hPCCA Treated Mice

In the PA patients, the target tissues are the liver and heart, and therefore, our initial survey was focused on hepatic and cardiac studies in mice treated with the AAV9-hPCCA vector. Using *[method]* with probes designed to detect the *PCCA* transgene and the endogenous *Gapdh* locus, copy numbers of the *PCCA* transgene were assessed at *[timepoints]* in heterozygous *Pcca*$^{+/-}$ and homozygous *Pcca* $^{-/-}$ animals dosed with AAV9-hPCCA at *[doses]*. As can be seen in ***Figure 9***, the AAV9-hPCCA genome is readily detected, indicating *[transduction results]*. Furthermore, the relative transduction between *Pcca*$^{-/-}$ mice and *Pcca*$^{+/-}$ *[is described in Figure 9]*.

Figure 9. Biodistribution of AAV9-hPCCA in Liver and Heart Tissues

[A cluster bar graph, providing the vector copy number of the PCCA transgene normalized to alleles of the Gapdh gene, as found in the liver and heart in Pcca-/- and Pcca+/- mice at different timepoints and doses.]

Vector copy number of the *PCCA* transgene in the liver and heart normalized to alleles of the *Gapdh* gene using *[method]* in the livers and hearts of AAV9-hPCCA treated *Pcca*$^{-/-}$ and *Pcca*$^{+/-}$ mice at *[timepoint]* and *[later timepoint]*-days post-treatment. Error bars are ± S.D.

5 ORPHAN DRUG STATUS

AAV9-hPCCA was designated (Designation request # DRU-2021-*[####]*) as an orphan drug or product for the treatment of patients with PA resulting from a deficiency of PCCA in the US by the FDA on September 27, 2021.

6 PATIENT SUBSET CONSIDERATIONS AND MEDICAL PLAUSIBILITY OF THE CHOSEN SUBSET

The prevalence of PA does not exceed 200,000. Therefore, subset considerations and medical plausibility of a subset are not applicable to the proposed indication of patients with PA resulting from a deficiency of PCCA in this request.

7 REGULATORY STATUS AND MARKETING HISTORY

NCATS is currently in late phase pre-clinical development of AAV9-hPCCA, where we have had an Initial Targeted Engagement for Regulatory Advice on CBER Products (INTERACT) meeting (submission #*[unique identifier]*) scheduled with FDA, Center for Biologics Evaluation and Research (CBER), Office of Tissues and Advanced Therapies (OTAT), on July 14, 2021, to discuss our completed proof of concept (POC) studies and planned IND enabling toxicology studies in preparation for IND submission fourth quarter of 2023.

AAV9-hPCCA is not currently marketed or approved for use in any country, including the United States. In accordance with section 526(a)(1) of the Federal Food, Drug, and Cosmetic Act [21 USC 360bb(a)(1)]; 21 CFR 316.20(b)(7); and 21 CFR 316.23(a), NCATS has not previously submitted a marketing application to FDA for the same active moiety in AAV9-hPCCA for the same rare disease or condition prior to submission of the AAV9-hPCCA Rare Pediatric Disease designation request.

8 DOCUMENTATION OF PATIENT POPULATION SIZE

PA is a rare disease in the US, which includes PCC enzyme deficiency from deleterious mutations in either the *PCCA* or *PCCB* genes. The *PCCA*- and *PCCB*- types appear to occur with equal distribution and have similar disease severity and manifestations.[5] There are no population-based prevalence studies, and most of the data used to estimate prevalence are from birth incidence mainly from studies conducted on NBS data identified in the medical literature. Overall, from these sources, PA in the US is a very low incidence/prevalence rare disorder, with birth incidence ranging from approximately 0.13-1.2 per 100,000 in the US, regardless of region or time period examined. Based on the annual US birth rate of 3.7 million/per year,[13] approximately 5-44 children are born in the US with both *PCCA*- and *PCCB*- related PA each year, with around 50% of individuals having *PCCA*-related PA (approximately 3-22 infants per year).

Studies identified from the medical literature are summarized as follows (see ***Table 4***):

- The most recent study was performed and published by Adhikari et al in 2020,[2] which reported the experience of the NBSeq project that evaluated whole-exome sequencing (WES)

as a new methodology for NBS. In this study, the authors obtained archived residual dried blood spots (DBS) and data for nearly all inborn errors of metabolism (IEM) cases (n=1,728 DBSs) identified using tandem mass spectrometry (MS/MS) from 4.5 million screened infants born in California between July 2005 and December 2013. They then compared the MS/MS with the results from WES. This was the largest study to date on sequencing efforts of an entire population of IEM-affected cases identified from NBS, inclusive of 48 different IEMs and the 78 genes associated with these disorders, representing 1,190 newborns: 805 IEM-affected newborns and 385 MS/MS false positives. There were 237 affected individuals with an organic acid disorder, of which 6 newborns were shown to have been diagnosed with PA, which approximates to a birth incidence of 0.13 per 100,000.

- Chapman et al, 2018,[3] conducted a study on NBS results for 3 disorders, maple syrup urine disease, PA, and methylmalonic aciduria (MMA), from 3 geographic regions around the world, including state screening labs in the US, the southwest region of Germany, and Kuwait. The US data was collected and analyzed from the US NBS data base from 1991 to 2000, and positive results from the diseases in questions were compared to the on-line reported birth rates per US states. Some states reported MMA and PA together, given the marker C3 being common to both. For PA alone, there were a total of 12 cases identified from a total of 2,912,901, for a birth incidence of 1:242,741, or 0.41 cases per 100,000. For MMA and PA together, there were 147 cases in 7,544,243 births, for a birth incidence of 1:50,709 (MMA alone birth incidence was 1:69,354, 3.5 times the incidence of PA alone).
- Almasi et al, 2019,[4] conducted a systematic literature review and meta-analysis on the worldwide epidemiology of PA, which estimated point prevalence of PA per 100,000 births calculated separately by region (North American, Europe, Asia-Pacific, Middle-East and North Africa (MENA)), and in 2 time periods 1981-2000, and 2001-present. They identified 43 studies included in the qualitative synthesis on epidemiology, and 31 studies included in the quantitative synthesis. The vast majority of articles reported on newborn screening programs (NBS) providing estimates on the birth prevalence of the disease, defined as the number of affected newborns divided by the total population screened, 8 of which described results from NBS programs in the US and are summarized in ***Table 4***. There were 6 studies used for the North America point estimate. The results showed birth prevalence ranged from 0.20 (California, US) to 1.35 (Ontario, Canada) per 100,000 newborns, with the pooled point estimate of 0.33 per 100,000 newborns in North America. Similar rates were noted in Europe, and higher rates in Japan and MENA. Pooled point estimates remained below 1 per 100,000 newborns in all regions except MENA, which were significantly higher (>3 per 100,000). The authors additionally noted a scarcity of studies in *PCCA*- and *PCCB*-subtypes but are reported to be approximately equally distributed,[7] and that broadly targeted population-based prevalence studies are not available. Their overall conclusions were that PA is an "ultra-rare" disorder; however, ultra-rare was not specifically defined.

- Of note, PA prevalence by ethnicity in the US was investigated in only one study, Feuchtbaum et al (2012),[14] who noted that only Native Americans were characterized by a significantly higher detection rate (6.7 per 100,000 newborns) than the overall rate (0.2 per 100,000) in the US.

Table 4: Brief Summaries of the US Studies Included in the Almasi Meta-Analysis[4]

Lead Author, Year of Publication	Estimated Birth Incidence per 100,000 Newborns	Brief Description
Feuchtbaum, 2012[14]	0.2	Study to describe birth prevalence of genetic disorders among different racial/ethnic groups through analysis of population-based NBS data from 2005-2010, inclusive of 2,282,138 newborns screened. Calculated birth incidence 0.2 per 100,000 newborns screened.
Frazier, 2006[15]	0.33	Report of North Carolina's 8-year experience with MS/MS NBS, and incidence of disease for selected amino acid, fatty acid and organic acid disorders, 1997-2005. Calculated incidence 1:300,000
Therrell, 2014[16]	0.42	Tabulation of 10-year NBS data for selected IEM from 2001-2011 from the National Newborn Screening Information System (NNSIS). Identified 105 PA cases in 25,026,374 newborns screened, for a birth incidence of 1:238,346 (0.42 per 100,000)
Weisfeld-Adams, 2009[17]	0.5	Retrospective DBS data analysis in patients with molecularly confirmed cobalamin C (cblcC) disease, in 4-year period (2005-2008) in NY. Total number of infants screened = 1,006,325, 5 cases of PA confirmed (1:201,265).
Comeau, 2004[18]	0.6	Study summarizes the New England Newborn Screening Program's (NENBSP) approach and experience, from Jan 1999- Jan 2003. PA clustered into panel testing, and not separately broken out within the paper. Birth incidence estimated to be around 0.6 per 100,000.
Naylor, 1999[19]	0.71	Study reporting results of NBS using MS/MS of more than 700,000 newborns from

Lead Author, Year of Publication	Estimated Birth Incidence per 100,000 Newborns	Brief Description
Naylor, 1999[18]	0.71	Study reporting results of NBS using MS/MS of more than 700,000 newborns from Pennsylvania, Ohio, North Carolina and Louisiana, which detected 163 IEMs. 32 patients were identified with organic acidemias, of which 5 had PA (0.71 per 100,000).
Chace, 2001[19]	~0.7	Study report on the validation of MS/MS analysis for MMA and PA from DBS obtained from 908,543 newborns from NBS from approximately 1992-2001. Results showed 1:64,896 with either MMA or PA by MS/MS, but birth incidence was not broken out by MMA or PA individually.
Zytkovicz, 2001[20]	1.2	Study summarizes 2-year MS/MS results for amino acid and fatty and organic acid disorders from DBS from the NENSP from Feb 1999 to Feb 2001, inclusive of 164,000 newborns. 36 infants were identified for C3, of which 2 were PA patients, for an estimated birth incidence of 1.2 per 100,000.

Thus overall, these findings are notable for the following:

First, there are no population-based prevalence studies for PA for the US population in the medical literature.

Second, all of the studies identified were based on birth incidence calculated from state NBS results in various regions around the US and in different time periods, mainly after 1999 when screening based on C3 levels detected by MS/MS became more broadly available. Birth incidence ranged from 0.13 to 1.2 per 100,000 newborns, and the results were largely similar across the US and over time. This translates to about 5-44 children born with PA per year, with 50% of these expected to be *PCCA*-type PA (3-22 cases per year). The most recent analysis conducted by Adhikari et al, 2020,[1] compared MS/MS with WES, which likely addressed false positives and is likely the most accurate. This study showed a birth incidence of 0.13 cases of PA per 100,000 newborns (6 cases per year), half of which are expected to be *PCCA*-type PA.

Third, based on the US Census Bureau's population count in 2020, there were 330,218,929 people living in the US.[5] This roughly translates to a PA prevalence ranging from, at the low end

resulting in premature death at a young age for many patients, the true prevalence is likely lower than this. Based on our good-faith assessment from expert opinion obtained from two of the world's experts on PA (Oleg A. Shchelochkov, MD and Charles P. Venditti, M.D., Ph.D., NIH, NHGRI), and from data from an NIH-conducted PA NHS,[8] we estimate there are ~50 *PCCA*-related PA patients living in the US.

In conclusion, the *PCCA*-related PA population amenable to therapy with AAV9-hPCCA gene therapy is a very low incidence/prevalence rare disease, with an estimated number of patients ranging from 50-3,992 patients in the US, which is far below the 200,000 prevalence cut-off to qualify for an Orphan designation.

9 RARE PEDIATRIC DISEASE DESIGNATION JUSTIFICATION

Based on the evidence presented above, pursuant to Section 908 of FDASIA,[1] and consistent with the Rare Pediatric Disease Priority Review Vouchers Guidance for Industry,[23] we believe that AAV9-hPCCA qualifies as a Rare Pediatric Drug Product for the treatment of *PCCA*-related PA. Specifically:

1) *PCCA*-type PA is a serious or life-threatening disease in which the serious or life-threatening manifestations primarily affect individuals aged from birth to 18 years.

Most PA patients are identified at birth through NBS. Despite this early recognition and initiation of best-available therapies, including dietary restrictions and supplements, and in some cases LT, severely affected patients continue to experience serious or life-threatening complications of their disease. The majority of patients will manifest irreversible serious end-organ damage, such as kidney, cardiac or CNS impairment, during childhood and adolescence, and there is a need for better treatments to prevent, mitigate or ameliorate these disease-related complications.

2) *PCCA*-related PA is a rare disease, with an estimated prevalence of fewer than 4,000 patients in the US.

We additionally note that AAV9-hPCCA is intended as a gene therapy specifically for gene replacement due to mutations in the PCC enzyme alpha subunit and has no foreseeable use in the treatment of any other disease or a different adult indication. AAV9-hPCCA is currently in late pre-clinical phases of testing at NIH, with planned progression to clinical trials within the next few years. The likely initial patient population will be children or adolescents.

We therefore request that AAV9-hPCCA be designated as a Rare Pediatric Disease Product for the treatment of the *PCCA*-related PA.

10 REFERENCES

1. FDASIA. Publ. L. 112-144. 126 Stat. 993. https://www.govinfo.gov/content/pkg/PLAW-112publ144/pdf/PLAW-112publ144.pdf. [Accessed February 3, 2022].

2. Adhikari AN, Gallagher RC, Wang Y, Currier RJ, Amatuni G, Bassaganyas L, Chen F, Kundu K, Kvale M, Mooney SD, Nussbaum RL, Randi SS, Sanford J, Shieh JT, Srinivasan R, Sunderam U, Tang H, Vaka D, Zou Y, Koenig BA, Kwok PY, Risch N, Puck JM, Brenner SE. The role of exome sequencing in newborn screening for inborn errors of metabolism. Nat Med. 2020 Sep;26(9):1392-1397. doi: 10.1038/s41591-020-0966-5.

3. Chapman KA, Gramer G, Viall S, Summar, ML. Incidence of maple syrup urine disease, propionic acidemia, and methylmalonic aciduria from newborn screening data. Mol Genet Metab Rep. 2018;15:106-09. https://doi.org/10.1016/j.ymgmr.2018.03.011.

4. Almasi T, Guey LT, Lukacs C, Csetneki K, Voko Z, Zelei T. Systematic literature review and meta-analysis on the epidemiology of propionic acidemia. Orphanet J Rare Dis. 2019;14:40. https://doi.org/10.1186/s13023-018-0987-z.

5. Shchelochkov OA, Carrillo N, Venditti C. Propionic Acidemia.2012 May 17 [updated 2016 Oct 6]. In: GeneReviews [Internet]. Seattle (WA): University of Washington, Seattle; 1993-2021. PMID: 22593918. https://www.ncbi.nlm.nih.gov/books/NBK92946/. [Accessed February 3, 2022]

6. Delgado C, Macias C, de la Sierra Garcia-Valdecasas M, Perez M, del Portal LR, Jimenez LM. Subacute presentation of propionic acidemia. J Child Neurol. 2007;22(12):1405-7. Doi: 10.1177/0883073807307080

7. Surtees RA, Matthews EE, Leonard JV. Neurologic Outcome of Propionic Acidemia. Pediatr Neurol. 1992;8:333-7. https://doi.org/10.1016/0887-8994(92)90085-D

8. Shchelochkov OA, Manoli I, Juneau P, Sloan JL, Ferry S, Myles J, Schoenfeld M, Pass A, McCoy S, Van Ryzin C, Wenger O, Levin M, Zein W, Huryn L, Snow J, Chlebowski C, Thurm A, Kopp JB, Chen KY, Venditti CP. Severity modeling of propionic acidemia using clinical and laboratory biomarkers. Genet Med. 2021;23(8):1534-42. doi: 10.1038/s41436-021-01173-2.

9. Chandler RJ, Chandrasekaran S, Carrillo-Carrasco N, Senac JS, Hofherr SE, Barry MA, Venditti CP. Adeno-associated virus serotype 8 gene transfer rescues a neonatal lethal murine model of propionic acidemia. Hum Gene Ther. 2011;22:477-81. doi: 10.1089/hum.2010.164.

10. Guenzel AJ, Hofherr SE, Hillestad M, Barry M, Weaver E, Venezia S, Kraus JP, Matern D, Barry MA. Generation of a hypomorphic model of propionic acidemia amenable to gene therapy testing. Mol Ther. 2013;21:1316-23. Doi: 10.1038/mt.2013.68.

11. Guenzel AJ, Hillestad ML, Matern D, Barry MA. Effects of adeno-associated virus serotype and tissue-specific expression on circulating biomarkers of propionic acidemia. Hum Gene Ther. 2014;25:837-43. Doi: 10.1089/hum.2014.012.

12. Guenzel AJ, Collard R, Kraus JP, Matern D, Barry MA. Long-term sex-biased correction of circulating propionic acidemia disease markers by adeno-associated virus vectors. Hum Gene Ther. 2015;26:153-60. Doi: 10.1089/hum.2014.126.

13. Centers for Disease Control and Prevention (CDC). National Center for Health Statistics, Births and Natality. Births: Final Data for 2019. https://www.cdc.gov/nchs/fastats/births.htm. [Accessed February 3, 2022]

14. Feuchtbaum L, Carter J, Dowray S, Currier RJ, Lorey F. Birth prevalence of disorders detectable through newborn screening by race/ethnicity. Genet Med. 2012;14:937-45. doi: 10.1038/gim.2012.76.

15. Frazier DM, Millington DS, McCandless SE, Koeberl DD, Weavil SD, Chaing SH, Muenzer J. The tandem mass spectrometry newborn screening experience in North Carolina: 1997–2005. J Inherit Metab Dis. 2006;29(1):76–85. Doi:10.1007/s10545-006-0228-9.

16. Therrell BL Jr, Lloyd-Puryear MA, Camp KM, Mann MY. Inborn errors of metabolism identified via newborn screening: Ten-year incidence data and costs of nutritional interventions for research agenda planning. Mol Genet Metab. 2014;113(1):14–26. Doi: 10.1016/j.ymgme.2014.07.009.

17. Weisfeld-Adams JD, Morrissey MA, Kirmse BM, Salveson BR, Wasserstein MP, McGuire PJ, Sunny S, Cohen-Pfeffer JL, Yu C, Caggana M, Diaz GA.. Newborn screening and early biochemical follow-up in combined methylmalonic aciduria and homocystinuria, cblC type, and utility of methionine as a secondary screening analyte. Mol Genet Metab 2010;99(2):116–123. doi: 10.1016/j.ymgme.2009.09.008.

18. Comeau AM, Larson C, Eaton RB. Integration of new genetic diseases into statewide newborn screening: New England experience. Am J Med Genet C Semin Med Genet. 2004;125C(1):35–41. doi: 10.1002/ajmg.c.30001.

19. Naylor EW, Chace DH. Automated tandem mass spectrometry for mass newborn screening for disorders in fatty acid, organic acid, and amino acid metabolism. J Child

Neurol 1999;14 Suppl 1:S4–S8. PMID:10593560. https://doi.org/10.1177%2F088307389901400102 1.

20. Chace DH, DiPerna JC, Kalas TA, Johnson RW, Naylor EW. Rapid diagnosis of methylmalonic and propionic acidemias: quantitative tandem mass spectrometric analysis of propionylcarnitine in filter-paper blood specimens obtained from newborns. Clin Chem. 2001;47(11):2040–4. PMID: 11673377. https://academic.oup.com/clinchem/article/47/11/2040/5639240

21. Zytkovicz TH, Fitzgerald EF, Marsden D, Larson CA, Shih VE, Johnson DM, et al. Tandem mass spectrometric analysis for amino, organic, and fatty acid disorders in newborn dried blood spots: a two-year summary from the New England newborn screening program. Clin Chem. 2001;47(11):1945 https://academic.oup.com/clinchem/article/47/11/1945/5639320

22. US Census Bureau. U S and World Population Clock https://www.census.gov/popclock/ [Accessed June 3, 2022]

23. FDA OOPD. Rare Pediatric Disease Priority Review Vouchers. Guidance for Industry. Draft Guidance, Revision 1. 2019. https://www.fda.gov/media/90014/download. [Accessed February 3, 2022].

APPENDIX A

Nucleotide Sequence of AAV9-hPCCA: The complete sequence of AAV9-hPCCA drug product, including the codon optimized human PCCA gene and the different AAV9 cassette elements with the corresponding ITRs and promoter.

[The complete sequence of the drug product]

Food and Drug Administration
10903 New Hampshire Avenue
WO32-5295
Silver Spring, MD 20993

National Center for Advancing Translational Sciences
9800 Medical Center Dr.
Rockville, MD 20850

Attention: *[Name and contact information for primary contact]*

Dear *[Primary Contact Name]*:

This letter acknowledges receipt of your rare pediatric disease[1] designation request for the following:

Name: Adeno-Associated Virus 9 human Propionyl-Coenzyme A (CoA) Carboxylase, alpha subunit (AAV9-hPCCA)

Disease or Condition: Treatment of patients with propionic acidemia (PA) resulting from a deficiency of Propionyl-CoA Carboxylase, alpha subunit (PCCA)

Date of request: June 17, 2022

Date of receipt: June 21, 2022

Designation request number: RPD-2022-*[###]*

Because you did not submit this request for rare pediatric disease designation at the same time as a request for orphan-drug designation or fast track designation, FDA is not under a 60-day deadline to respond (see section 529(d)(2) and (3) of the Federal Food, Drug, and Cosmetic Act (21 U.S.C. 360ff(d)(2) and (3))). However, if you did submit an orphan-drug designation and/or fast track request in the appropriate timeframe under the statute and you think the 60-day deadline should apply, you must notify OOPD immediately. Regardless, we will aim to respond to your request in a timely manner.

Note that we cannot accept your request for designation consideration if your marketing application for this drug for this disease or condition has already been filed. Please let us

[1] "Rare pediatric disease" is defined in section 529(a)(3) of the Federal Food, Drug, and Cosmetic Act (21 U.S.C. 360ff(a)(3)).

know promptly if your marketing application has already been filed by calling calling *[phone number]* or alternatively *[phone number]*. In such case, you may still receive a rare pediatric disease priority review voucher if you requested such a voucher in your original marketing application and you meet the eligibility criteria described in section 529 of the FD&C Act.

Your RPD designation request will be reviewed by both the Office of Orphan Products Development (OOPD) and the Office of Pediatric Therapeutics (OPT). OOPD will be determining if the disease identified is rare, within the meaning of section 526 of the Federal Food, Drug, and Cosmetic Act (21 U.S.C. 360bb), and OPT will be determining if the disease is a serious or life-threatening disease in which the serious or life-threatening manifestations primarily affect individuals aged from birth to 18 years. You may be contacted by either office if there are questions about your request.

OOPD will respond to your request for designation by email. Please provide updated contact information if and when it changes. We will assume that all emails from your representatives, or email addresses provided as a point of contact in your request, are FDA secure when responding to those email addresses. Transmissions to and from the FDA using FDA secure email addresses are encrypted. You can establish a secure email address link to FDA by sending a request to *[email address]*. There may be a fee to a commercial enterprise for establishing a digital certificate as part of the set-up process before emails can be sent to FDA encrypted.

All communications to OOPD concerning your request should be identified with the above designation request number. Should you have any questions regarding whether the disease is a rare disease or condition within the meaning of section 526 of the FD&C Act, please contact *[name of FDA official]* at *[phone number]* or alternatively at *[phone number]*.

Should you have any questions regarding whether the disease is a "serious or life-threatening disease in which the serious or life-threatening manifestations primarily affect individuals aged from birth to 18 years," please contact *[name of FDA official]* at *[phone number]*.

Sincerely yours,
[Digital signature of FDA official]

[Name and title of FDA Official]
Office of Orphan Products Development

Food and Drug Administration
10903 New Hampshire Avenue
WO32- 5295
Silver Spring, MD 20993

National Institutes of Health (NIH)
National Center for Advancing Translational Sciences (NCATS)
9800 Medical Center Dr.
Rockville, MD 20850

Attention: *[Name and contact information for primary contact]*

Re:Designation request #RPD-2022-*[###]*
Dated: June 17, 2022
Received: June 21, 2022

Dear *[Primary Contact Name]*:

This letter responds to your request for rare pediatric disease designation of Adeno-Associated Virus 9 human Propionyl-Coenzyme A (CoA) Carboxylase, alpha subunit (AAV9-hPCCA) for treatment of "propionic acidemia (PA) resulting from a deficiency of Propionyl-CoA Carboxylase, alpha subunit (PCCA)."

We hereby grant your request and designate Adeno-Associated Virus 9 human Propionyl-Coenzyme A (CoA) Carboxylase, alpha subunit (AAV9-hPCCA) for *treatment of propionic acidemia (PA) resulting from a deficiency of Propionyl-CoA Carboxylase, alpha subunit (PCCA)* as a drug for a "rare pediatric disease," as defined in section 529(a)(3) of the Federal Food, Drug, and Cosmetic Act (FD&C Act) (21 U.S.C. 360ff(a)(3)). We determined that PA resulting from a deficiency in PCCA meets the definition of a rare pediatric disease based on the information you submitted and additional information from the literature.

Based on information you provided and additional information from the literature, there is sufficient information to demonstrate that acute metabolic decompensation, growth impairment, failure to thrive, neurological impairment, developmental delays/cognitive impairment, pancreatitis, cardiomyopathy, conduction abnormalities, optic atrophy, and hearing loss are serious or life-threatening manifestations of PA resulting from a deficiency in PCCA that primarily affect children. PA resulting from a deficiency in PCCA also meets the definition of "rare disease or condition" at section 526 of the FD&C Act. Therefore,

your drug is eligible for rare pediatric disease designation for treatment of PA resulting from a deficiency in PCCA.

The statute also requires that FDA, in responding to rare pediatric disease designation requests, decide whether the associated marketing application for the drug will be a "rare pediatric disease product application." Section 529(d)(3) of the FD&C Act (21 U.S.C. 360ff(d)(3)). At this time, we cannot designate any associated marketing application for the drug as a "rare pediatric disease product application," as defined in section 529(a)(4) of the FD&C Act (21 U.S.C. 360ff(a)(4)), because we cannot assess whether it will qualify as such until the time of approval or licensure. We can only conditionally designate the marketing application as a "rare pediatric disease product application" *pending the final determination at the time of approval or licensure whether the application meets all of the eligibility criteria set forth in section 529(a)(4) of the FD&C Act.* We strongly recommend that you consult with the applicable review division early during your development program to address any questions that you may have about the criteria noted below for a rare pediatric disease product application that will be eligible for a rare pediatric disease priority review voucher:

- It is a human drug application as defined in section 735(1) of the FD&C Act[1] that:
    - Is for a drug or biological product that is for the prevention or treatment of a rare pediatric disease;
    - Is for—
        - such a drug
            - that contains no active moiety (as defined by the Secretary in 21 CFR 314.3 (or any successor regulations)) that has been previously approved in any other application under subsection (b)(1), (b)(2), or (j) of section 505 of the FD&C Act; and
            - that is the subject of an application submitted under section 505(b)(1) of the FD&C Act; or
        - such a biological product—
            - that contains no active ingredient that has been previously approved in any other application under section 351(a) or 351(k) of the Public Health Service Act (PHS Act); and
            - that is the subject of an application submitted under section 351(a) of the PHS Act.
    - FDA deems eligible for priority review.

[1] This definition includes drugs and most biological drugs, including vaccines, but excludes blood components and certain other biological drug products. For details, refer to section 735(1) of the FD&C Act (21 U.S.C. 379g(1)). The definition does not cover medical devices.

- Relies on clinical data derived from studies examining a pediatric population and dosages of the drug intended for that population.

- Does not seek approval for an adult indication in the original rare pediatric disease product application.

- Is submitted on or after October 7, 2012 and approved after September 30, 2016.

The final answer to whether your marketing application qualifies as a "rare pediatric disease product application" as defined by statute will come in the form of an award or non-award of a rare pediatric disease priority review voucher at the time of marketing approval, should you request such a voucher in your marketing application.

If you are interested in receiving such a voucher, you must include a voucher request in your original marketing application to FDA for the rare pediatric disease product. This voucher request should include a copy of this designation letter to show that your drug is for a "rare pediatric disease" and explain how your application meets the remaining eligibility criteria in section 529(a)(4) of the FD&C Act (21 U.S.C. 360ff(a)(4)). Please notify us (1) when you submit a marketing application that includes a voucher request, and (2) if, upon approval of your marketing application, you receive a voucher. Please also provide a copy of any voucher transfer notification that you provide to the appropriate FDA review division. We encourage early communication with the relevant review division as to your interest in receiving a priority review voucher to allow discussion of the issues.

Please notify us whenever there is a change in the contact information provided in your designation request, or there is a change in ownership of the designated product. Cite the designation request number in all your correspondence.

Should you have any questions specifically about the disease being a "serious or life-threatening disease in which the serious or life-threatening manifestations primarily affect individuals aged from birth to 18 years," please contact *[name of FDA official]* at *[phone number]*.

Should you have any other questions about this rare pediatric disease designation, please contact *[name of FDA official]* at *[phone number]* or alternatively at *[phone number]*.

Congratulations on obtaining your rare pediatric disease designation.

Sincerely yours,

[Digital signature of FDA official]

[FDA official name and title]
Office of Pediatric Therapeutics

[Digital signature of FDA official]

[Name and title of FDA official]
Office of Orphan Products Development
